# HD6277 Suppresses Muscle Atrophy by Promoting Myogenic Factors and Inhibiting Proteolysis in Aged Mice

**DOI:** 10.1002/jcsm.13805

**Published:** 2025-04-14

**Authors:** Joo Won Kim, SukHwan Yun, Min Jeong Park, Eyun Song, Sooyeon Jang, Ahreum Jang, Kyung Mook Choi, Sei Hyun Baik, Hwan‐Jin Hwang, Hye Jin Yoo

**Affiliations:** ^1^ BK21 Graduate Program, Department of Biomedical Sciences Korea University College of Medicine Seoul Republic of Korea; ^2^ Division of Endocrinology and Metabolism, Department of Internal Medicine Korea University College of Medicine Seoul Republic of Korea

**Keywords:** GPR40, muscle atrophy, myoblast, myogenic factors, myotube, sarcopenia

## Abstract

**Background:**

G protein–coupled receptor 40 (GPR40) acts as a modulator of various physiological functions, including glycaemic lowering, anti‐inflammation and antioxidative stress, in several tissues. However, the role of GPR40 in skeletal muscles remains unclear.

**Methods:**

To investigate the roles of muscle GPR40, C2C12 myoblasts and myotubes were stimulated with palmitate and HD6277, a GPR40 agonist. Muscle strength and myofiber thickness were measured in obese and aged mice fed HD6277.

**Results:**

In C2C12 myoblasts, the addition of HD6277 induced phosphorylated Akt levels and expression of the myogenic factors, myogenin (MyoG), myocyte enhancer factor 2C (Mef2c) and myosin heavy chain (MyHC, *p* < 0.05). These changes resulted in accelerated muscle differentiation from myoblasts to myotubes (MyHC‐positive area +56.52%; myotube width +34.08% vs. Veh, *p* < 0.05). In C2C12 myotubes, a palmitate‐mediated decrease in the phosphorylation of forkhead box protein O1A (FOXO1A) and increase in the expression of E3 ubiquitin ligases, atrogin‐1 and muscle RING‐finger protein 1 (MuRF1) were reversed by HD6277 (*p* < 0.05). Additionally, HD6277 inhibited palmitate‐induced apoptotic events such as the Bcl‐2 (Bcl2)‐associated X protein (Bax)/Bcl‐2 ratio, caspase 3 cleavage and nuclear fragmentation in C2C12 myoblasts and myotubes (*p* < 0.05). These beneficial HD6277‐mediated actions disappeared after the addition of an Akt inhibitor (*p* < 0.05). Similar to in vitro studies, HD6277 administration in obese and aged mice increased myogenic factors and decreased E3 ubiquitin ligase expression and apoptotic events (*p* < 0.05). HD6277 increased muscle strength (+9.88% vs. Aged, *p* < 0.05) and myofiber thickness (+29.01% vs. Aged, *p* < 0.05) in aging mice but only improved myofiber thickness (+11.84% vs. HFD, *p* < 0.05) in obese mice.

**Conclusion:**

HD6277 can increase myogenic factors and reduce E3 ligase‐mediated proteolysis to inhibit muscle atrophy in aged mice. Our results suggest that GPR40 agonists may have potential as therapeutic agents for sarcopenia.

## 1 Introduction

Sarcopenia refers to excessive loss of skeletal muscle mass and strength. It is associated with poor physical performance, development of cardiovascular diseases (CVD), such as atherosclerosis, and mortality in older adults [1]. Obesity and diabetes play a crucial role in sarcopenia by accelerating muscle fibre atrophy through persistent inflammation and oxidative stress [2]. Increased reactive oxygen species (ROS) cause muscular proteolysis, fat infiltration and declined regenerative capacity, resulting in lower muscle quality [3]. Resistance training and nutritional interventions are considered in the treatment of sarcopenia, but their benefits are limited in the elderly due to anorexia and low physical capacity [4, 5]. Therefore, pharmacological approaches to reduce muscle fibre atrophy are needed urgently.

Muscle fibre atrophy is strongly associated with reduced muscle regeneration and enhanced proteolysis. Muscle regeneration, or myogenesis, is the process of repairing and growing multinucleated myofibers in damaged skeletal muscles [6]. The loss of satellite cells, myogenic stem cells and changes in the energy metabolism of muscle fibres lead to a decline in myogenic capacity, which can be accelerated by aging [3, 7]. Muscle proteolysis, which involves the breakdown of myofibrillar proteins, is regulated by E3 ubiquitin ligases and cell death signalling. During aging, persistent and excessive oxidative stress enhances the ubiquitin–proteasome system and caspase 3 activity [3]. Oxidative stress also reduces protein synthesis by inducing pro‐inflammatory cytokines and mitochondrial dysfunction, which leads to decreased muscle mass and strength [3]. Therefore, both muscle regeneration and proteolysis are considered therapeutic targets for sarcopenia.

G protein–coupled receptor 40 (GPR40) has emerged as a key molecule for resolving several metabolic stimuli, including hyperglycaemia and oxidative stress. In the pancreas, after recognition by long‐chain free fatty acids (FFAs), GPR40 can rapidly couple with G protein subunit alpha q (Gαq), induce calcium (Ca^2+^) influx into cells and amplify glucose‐stimulated insulin secretion (GSIS) [8]. Serum levels of glucagon‐like peptide 1 (GLP‐1) and gastric inhibitory polypeptide (GIP), both of which induce insulin secretion from the pancreas via their respective receptors, can be modulated by intestinal GPR40/Gαs/cyclic adenosine monophosphate (cAMP) signalling [9, 10]. Therefore, many GPR40‐specific small chemicals are being developed and modified for the treatment of type 2 diabetes (T2DM) [11]. Although most research on GPR40 has focused on its hypoglycaemic effects, there is also growing evidence that it has protective actions against other metabolic disorders. Verma et al. reported that pancreatic GPR40 activation inhibited pro‐inflammatory cytokine‐induced ROS production, caspase 3 activity and reduction of adenosine triphosphate (ATP) synthesis in a cAMP‐dependent manner [12]. In liver tissues, GPR40 agonism is involved in suppressing excessive lipid accumulation and maintaining glucose homeostasis in a Gαq/Ca^2+^/AMP kinase–dependent manner [13, 14]. Furthermore, our previous reports have shown that GPR40 activation reversed lipopolysaccharide (LPS)‐induced nuclear factor kappa B (NFkB) phosphorylation and adhesion molecule expression and palmitate‐mediated endoplasmic reticulum (ER) stress and cytotoxicity in vascular endothelial cells in a nuclear factor erythroid 2–related factor 2 (NRF2)/haem oxygenase‐1 (HO‐1)–dependent manner [15, 16]. GPR40 is also expressed in muscle [17], but its benefits in muscle have not yet been identified. Therefore, we investigated the protective roles of GPR40 agonism against muscle atrophy in the present study.

This study aims to determine the beneficial roles of GPR40 agonism against muscle atrophy in obese and aged mice. We assessed whether treatment with the GPR40 agonist, HD6277, (i) induces myotube formation, (ii) reduces muscular proteolysis and (iii) inhibits cytotoxicity in palmitate‐ or H_2_O_2_‐stimulated C2C12 cells. Furthermore, we measured whether GPR40 administration (iv) improves muscle mass and strength in obese and aged mice.

## 2 Methods

### 2.1 Cell Culture and Reagents

C2C12 myoblasts were obtained from ATCC (VA, USA) and cultured in Dulbecco's modified eagle medium (DMEM; Thermo Fisher Scientific, MA, USA) supplemented with 10% fetal bovine serum (FBS; Thermo Fisher Scientific), 50‐U/mL penicillin and 50‐μg/mL streptomycin (Thermo Fisher Scientific) at 37°C. To induce differentiation from myoblasts to myotubes, DMEM containing 2% horse serum (Thermo Fisher Scientific) was replaced when the myoblast population reached approximately 80%–90%. We obtained fully differentiated C2C12 myotubes after a 5‐day differentiation period. GPR40 agonists (Hyundai Pharm Ltd., Seoul, Republic of Korea), GPR40 antagonist GW1100 (Cayman Chemical, MI, USA) and an Akt inhibitor (1L6‐hydroxymethyl‐chiro‐inositol‐2‐(*R*)‐2‐*O*‐methyl‐3‐*O*‐octadecyl‐sn‐glycerocarbonate; Sigma‐Aldrich, MO, USA) were dissolved in dimethyl sulfoxide (DMSO, Sigma‐Aldrich). Palmitic acid (Sigma‐Aldrich) was dissolved in ethanol (Merck Millipore, MA, USA) and conjugated with bovine serum albumin (BSA; Thermo Fisher Scientific).

### 2.2 Mouse Model and Drug Administration

Male 8‐week‐old C57BL/6 mice were obtained from OrientBio (SeongNam, Republic of Korea) and divided into three groups, as follows: normal diet (ND)–fed group (*n* = 7), 45% high‐fat diet (HFD)–fed group (*n* = 7) and HFD mixed with 0.02% HD6277 (about 20–30 mg/kg/day)‐fed group (*n* = 7). After 10 weeks, tibialis anterior (TA) muscles were harvested. Male 19‐month‐old mice were purchased from Janvier Labs (Le Genest‐Saint‐Isle, France) and divided into two groups; ND‐fed group (*n* = 7) and ND mixed with 0.02% HD6277 (about 30–35 mg/kg/day)‐fed group (*n* = 9). After 21 weeks, their TA muscles were harvested. All mice were maintained on a 12‐h light/dark cycle and had ad libitum access to feed and water. We measured the food intake and body weight of the mice every week. This study was approved by the Institutional Animal Care and Use Committee (IACUC) at Korea University, Seoul, Korea.

### 2.3 Western Blotting

The total proteins were extracted from C2C12 cells and muscle tissues, separated by sodium dodecyl sulfate–polyacrylamide gel electrophoresis (SDS‐PAGE) and transferred to a nitrocellulose (NC) membrane (Amersham Bioscience, MA, USA). The membranes were soaked sequentially in blocking solution (5% non‐fat dry milk or 5% BSA dissolved in Tris‐buffered saline with 0.05% Tween 20), blocking solution mixed with primary antibody and blocking solution mixed with horseradish peroxidase (HRP)–conjugated secondary antibodies (Vector Laboratories, CA, USA). Enhanced chemiluminescence (ECL) kits (Bio‐Rad, Hercules, CA, USA) were used to detect HRP‐conjugated bands. The density of the bands sensitized on the X‐ray film (Agfa, Mechelen, Belgium) was measured using the ImageJ program (National Institutes of Health, MD, USA). The antibodies used in the present study are listed in Table S1.

### 2.4 Quantitative Real‐Time PCR (qPCR)

Total RNA was isolated from C2C12 myoblasts and muscle tissues using Qiazol Lysis Reagent (Qiagen, Hilden, Germany) and used to synthesize complementary deoxyribonucleic acid (cDNA). The transcript levels were determined using the synthesized cDNA and GreenStar qPCR PreMix (Bioneer Corporation, Daejeon, Republic of Korea) and normalized to beta‐actin. The primers for qPCR analysis are shown in Table S2.

### 2.5 Myosin Heavy Chain (MyHC) Staining

C2C12 cells that were differentiated for 2 days were fixed in 3.7% formaldehyde for 10 min at room temperature and permeabilized in prechilled methanol for 10 min at −20°C. The permeabilized cells were sequentially incubated with blocking solution, blocking solution mixed with MyHC antibody and blocking solution mixed with FITC‐conjugated secondary antibody. The nuclei were stained with Hoechst solution. The images for stained cells were obtained with a fluorescence microscope (Thermo Fisher Scientific). The MyHC‐positive area and myotube thickness were measured in at least six images per group using the ImageJ program (National Institutes of Health).

### 2.6 Terminal Deoxynucleotidyl Transferase dUTP Nick End Labelling (TUNEL) Assay

C2C12 myoblasts and myotubes were stained using a TUNEL assay kit (MyBioSource, San Diego, CA, USA) according to the manufacturer's instructions to visualize DNA fragmentation. The images for stained cells were obtained with a fluorescence microscope (Thermo Fisher Scientific). The ratio of TUNEL‐positive cells (red fluorescence) to total cells (blue fluorescence) was counted in six views per group.

### 2.7 Cell Viability Assay

To measure cell viability, C2C12 myoblasts and myofibers were stimulated with EZ‐Cytox solution (DozenBio, Seoul, Korea) according to the user manual. The optical density (OD) absorbance was measured at a 450‐nm wavelength using a microplate reader (Bio‐Rad Laboratories).

### 2.8 Grip Strength Test

Four‐limb grip strength was measured five times in triplicate using a grip strength meter (Bioseb, Vitrolles, France). When the mice gripped the square‐grid bar of the grip strength meter with their four limbs, we gently pulled back until the mice released their limbs; the grip strength meter calculated the maximum pulling force. The grip strength test was conducted in all animals a week before they were sacrificed.

### 2.9 Measurement of Muscle Fibre Cross‐Sectional Area (CSA)

Muscles were fixed in 3.7% formaldehyde, embedded in paraffin and cut into 4‐μm sections. The sections were sequentially deparaffinized, rehydrated and stained with haematoxylin and eosin (H&E) dye according to the user manuals. The stained sections were observed under a microscope (Olympus, Tokyo, Japan). The CSA of muscle fibres was measured in five randomly selected views per sample using the ImageJ program (National Institutes of Health).

### 2.10 Statistical Analysis

**FIGURE 1:**
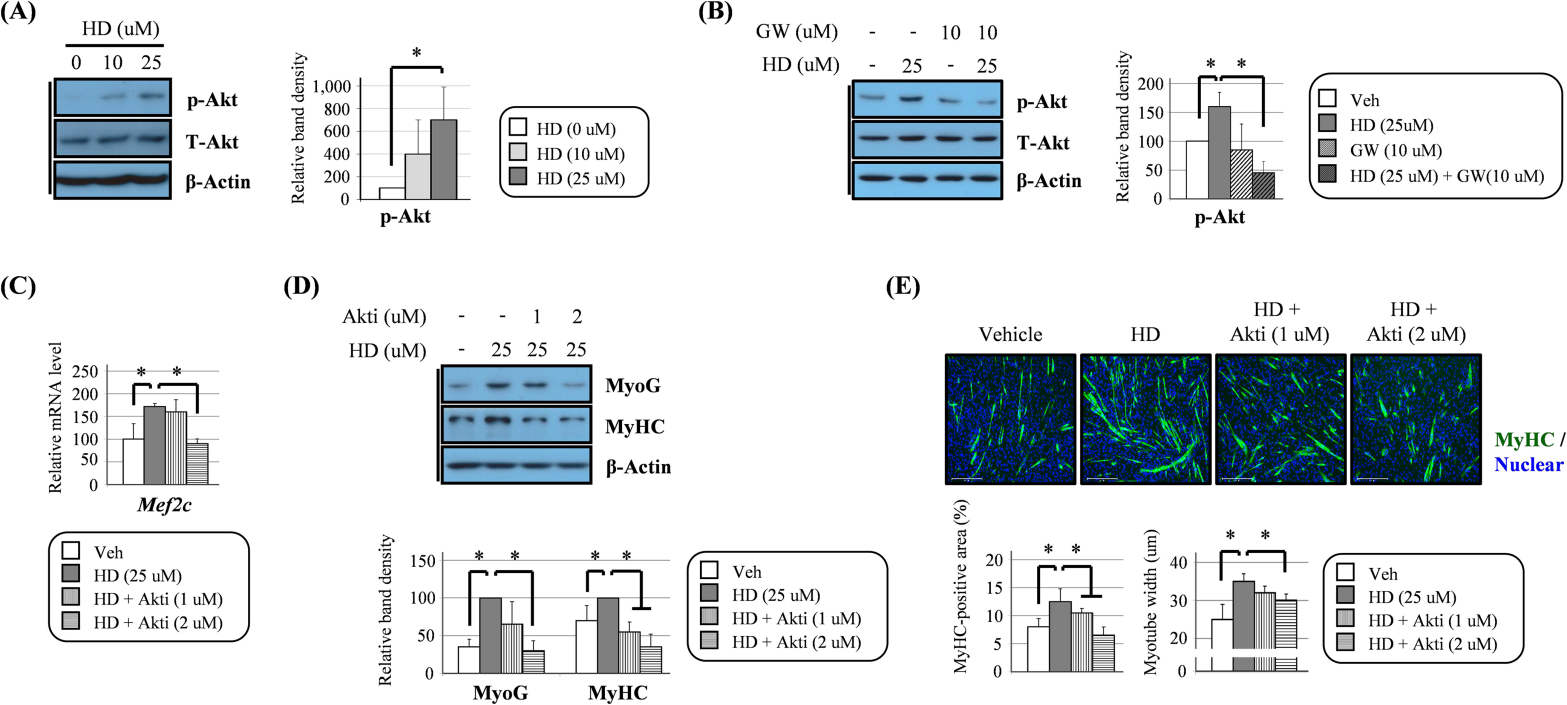
HD6277 accelerated myotube formation in an Akt‐dependent manner in C2C12 myoblasts. (A,B) The cells were differentiated with various doses of HD6277 (0, 10 or 25 μM) or HD6277 plus GW1100 (10 μM) for 2 days, and then, the phosphorylated Akt level was determined by Western blotting. (C) The cells were differentiated with HD6277 (25 μM) with or without an Akt inhibitor (1 or 2 μM) for 2 days, and then, the transcript level of Mef2c was analysed by qPCR. (D) MyoG and MyHC levels were determined by Western blotting. (E) The MyHC‐positive area and myotube width were calculated in MyHC‐stained cells (green fluorescence). Scale bar: 275 μm. Akti, Akt inhibitor; GW, GW1100; HD, HD6277; Veh, vehicle. Error bars indicate the mean ± SD (*p < 0.05; ANOVA with post hoc *t* test).

**FIGURE 2:**
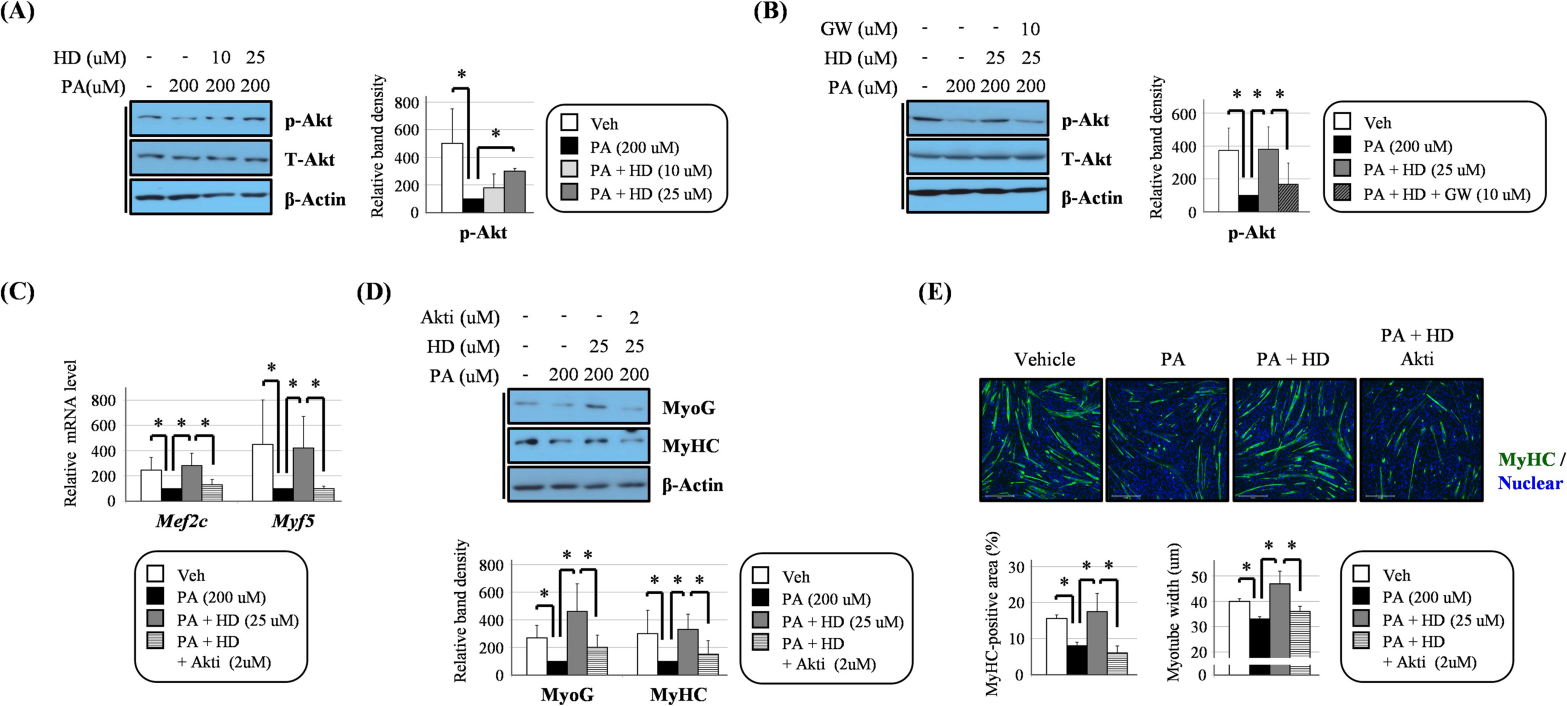
HD6277 inhibited the palmitate‐mediated reduction in myotube formation in an Akt‐dependent manner in C2C12 myoblasts. (A,B) The cells were stimulated with palmitate (200 μM), palmitate plus HD6277 (10 or 25 μM) or palmitate plus HD6277 with GW1100 (10 μM). The phosphorylated Akt level was analysed by Western blotting. (C) The cells were stimulated with palmitate (200 μM), palmitate plus HD6277 (25 μM) or palmitate plus HD6277 with Akt inhibitor (2 μM) and then differentiated for 2 days. The transcript levels of Mef2c and Myf5 were analysed by qPCR. (D) MyoG and MyHC levels were analysed by Western blotting. (E) MyHC‐stained cells (green fluorescence) were observed under a fluorescence microscope to calculate the MyHC‐positive area and myotube width. Scale bar: 275 μm. Akti, Akt inhibitor; GW, GW1100; HD, HD6277; PA, palmitate; Veh, vehicle. Error bars indicate the mean ± SD (*p < 0.05; ANOVA with post hoc *t* test).

**FIGURE 3:**
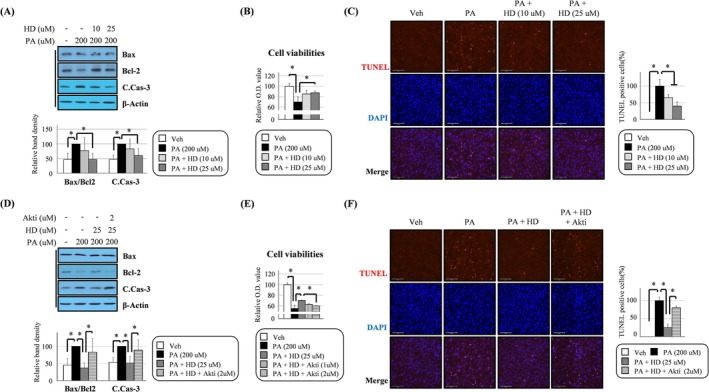
HD6277 suppressed palmitate‐mediated cytotoxicity in an Akt‐dependent manner in C2C12 myoblasts. (A,D) The cells were stimulated with palmitate (200 μM), palmitate plus HD6277 (10 or 25 μM) or palmitate plus HD6277 with Akt inhibitor (1 or 2 μM), after which the Bax/Bcl2 ratio and cleaved caspase 3 level were determined by Western blotting. (B,E) Cell viability was measured using the EZ‐Cytox solution. (C,F) DNA fragmentation was visualized using a TUNEL assay kit. Scale bar: 125 μm. TUNEL‐positive cells (red fluorescence) were observed and counted under a fluorescence microscope. Akti, Akt inhibitor; C.Cas‐3, cleaved caspase 3; HD, HD6277; PA, palmitate; Veh, vehicle. Error bars indicate the mean ± SD (*p < 0.05; ANOVA with post hoc *t* test).

**FIGURE 4:**
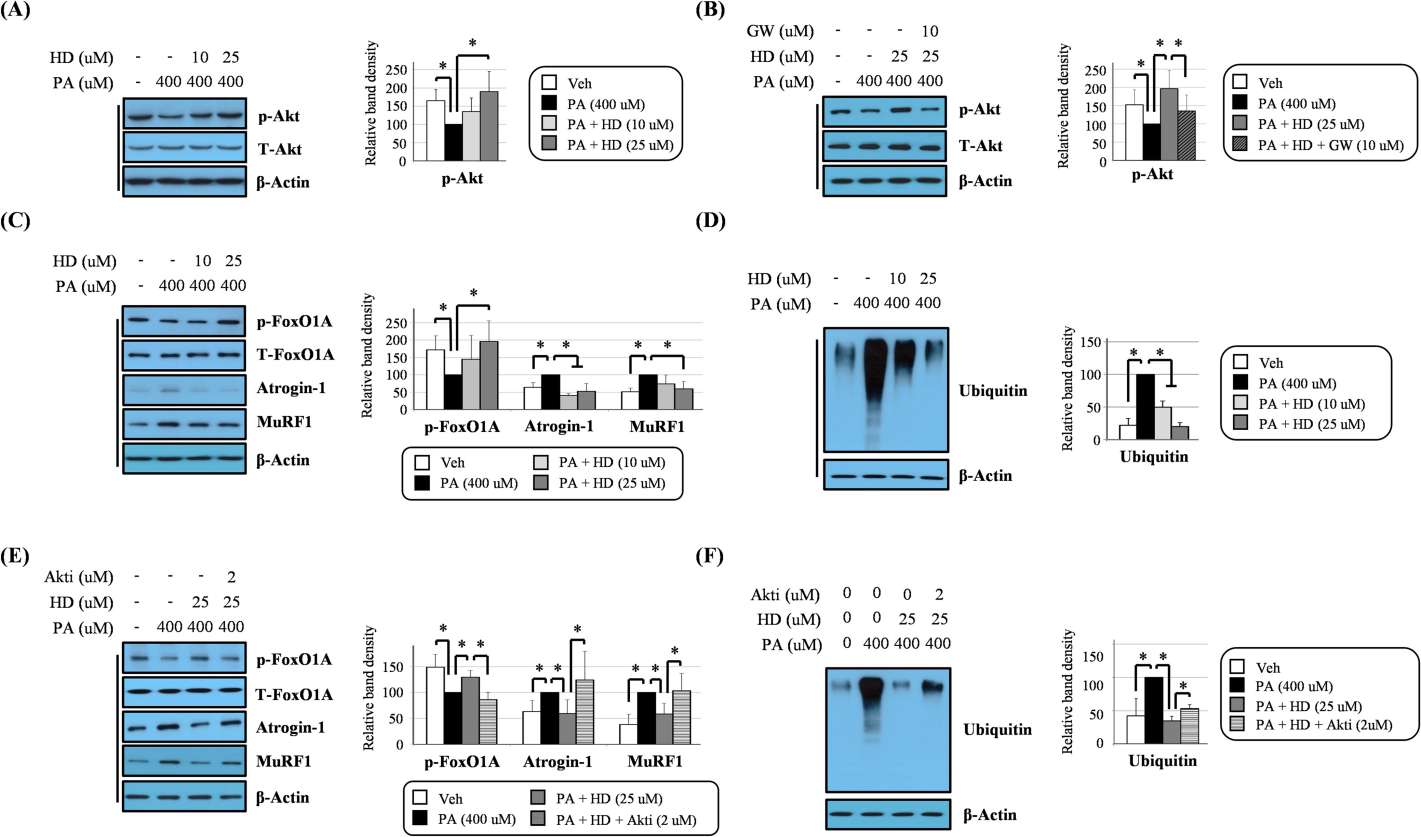
HD6277 reduced the palmitate‐induced proteolytic processes in an Akt‐dependent manner in C2C12 myotubes. (A,B) Fully differentiated cells were stimulated with palmitate (400 μM), palmitate plus HD6277 (10 or 25 μM) or palmitate plus HD6277 with GW1100 (10 μM), and then, the phosphorylated Akt level was determined by Western blotting. (C–F) Fully differentiated cells were stimulated with palmitate (400 μM), palmitate plus HD6277 (10 or 25 μM) or palmitate plus HD6277 with Akt inhibitor (2 μM). Western blotting was then performed to analyse the levels of phosphorylated FOXO1A, atrogin‐1, MuRF1 and ubiquitination. Akti, Akt inhibitor; GW, GW1100; HD, HD6277; PA, palmitate; Veh, vehicle. Error bars indicate the mean ± SD (*p < 0.05; ANOVA with post hoc *t* test).

**FIGURE 5:**
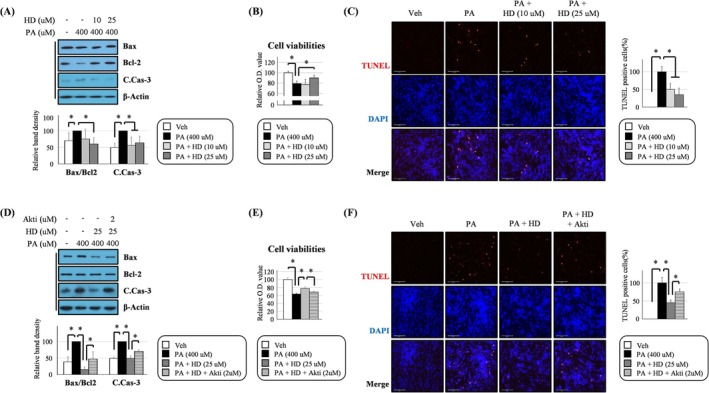
HD6277 inhibited palmitate‐induced cytotoxicity in an Akt‐dependent manner in C2C12 myotubes. (A,D) Fully differentiated cells were incubated with palmitate (400 μM), palmitate plus HD6277 (10 or 25 μM) or palmitate plus HD6277 with Akt inhibitor (2 μM), and then, Western blotting was performed to determine the levels of the Bax/Bcl2 ratio and cleaved caspase 3. (B,E) Cell viability was measured using EZ‐Cytox solution. (C,F) DNA fragmentation was analysed by the TUNEL assay. Scale bar: 125 μm. TUNEL‐positive cells (red fluorescence) were calculated under a fluorescence microscope. Akti, Akt inhibitor; C.Cas‐3, cleaved caspase 3; HD, HD6277; PA, palmitate; Veh, vehicle. Error bars indicate the mean ± SD (*p < 0.05; ANOVA with post hoc *t* test).

**FIGURE 6:**
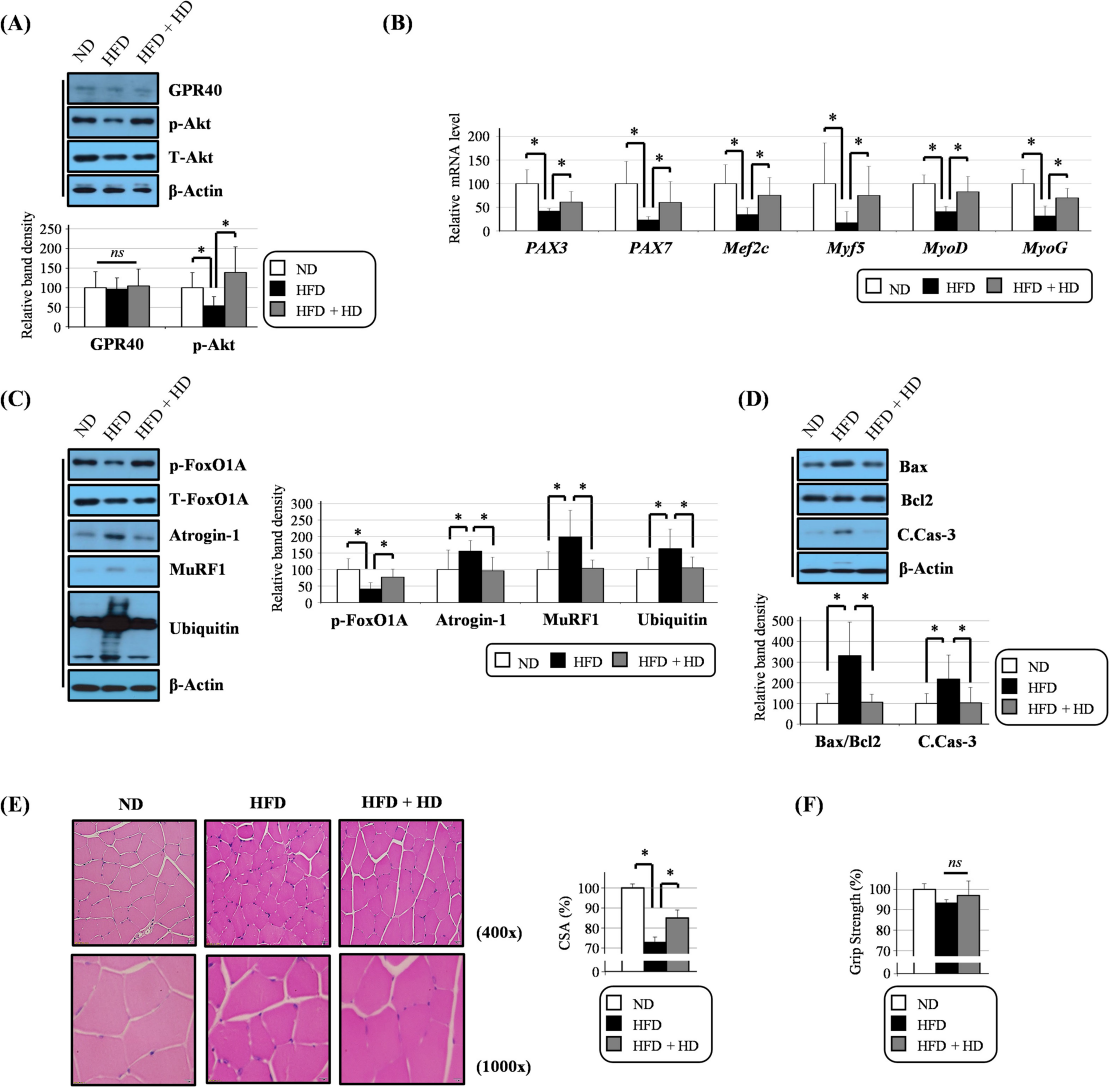
HD6277 induced the transcription of myogenic genes and reduced the expression of proteolytic proteins in obese mice. (A) The phosphorylated Akt levels were determined by Western blotting. (B) Transcript levels of PAX3, PAX7, Mef2c, Myf5, MyoD and MyoG were calculated by qPCR. (C,D) Western blotting was performed to analyse the levels of phosphorylated FOXO1A, atrogin‐1, MuRF, ubiquitination, Bax, Bcl2 and cleaved caspase 3. (E) The cross‐sectional area (CSA) of muscle fibres was measured in images stained with H&E dyes. Scale bar: 20 (400×) and 10 μm (1000×). (F) A grip strength device was used to determine four‐limb muscle strength. C.Cas‐3, cleaved caspase 3; HFD, high‐fat diet–fed mice; HFD + HD, HFD mixed with HD6277‐fed mice; ND, normal diet–fed mice; *ns*, no significant. Error bars indicate the mean ± SD (*p < 0.05; ANOVA with post hoc *t* test).

**FIGURE 7:**
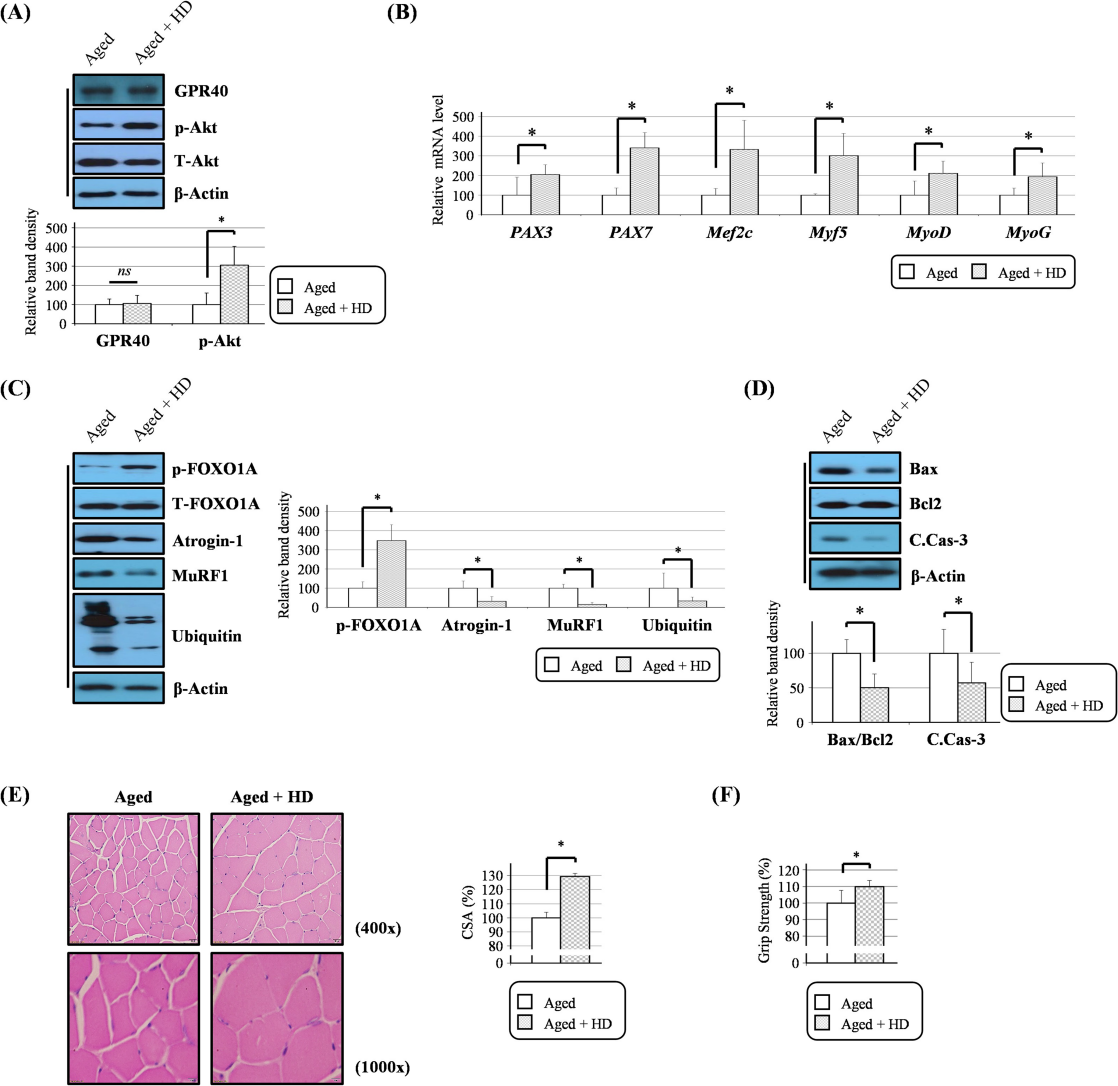
HD6277 delayed muscle atrophy in aged mice. (A) Western blotting showed the phosphorylated Akt level. (B) PAX3, PAX7, Mef2c, Myf5, MyoD and MyoG transcripts were determined by qPCR. (C,D) The levels of phosphorylated FOXO1A, atrogin‐1, MuRF, ubiquitination, Bax, Bcl2 and cleaved caspase 3 were analysed by Western blotting. (E) The CSA of the muscle fibres was calculated in images stained with H&E dyes. Scale bar: 20 (400×) and 10 μm (1000×). (F) The grip strength device was used to analyse four‐limb muscle strength. Aged, normal diet–fed aged mice; Aged + HD, normal diet mixed with HD6277‐fed aged mice; C.Cas‐3, cleaved caspase 3. Error bars indicate the mean ± SD (*p < 0.05; two‐tailed *t* tests assuming unequal variance).

Differences between the three groups were analysed for statistical significance using analysis of variance (ANOVA) with a post hoc *t* test (Figures 1, 2, 3, 4, 5, 6). For comparison between two groups, two‐tailed *t* tests assuming unequal variance were used to evaluate differences (Figure 7). All graphs presented the mean ± standard deviation (SD). Results were considered significant at *p* < 0.05.

## 3 Results

### 3.1 HD6277 Accelerated Myotube Formation in an Akt‐Dependent Manner in C2C12 Myoblasts

Western blotting showed that HD6277, a GPR40 agonist, increased Akt phosphorylation (Figure 1A). This effect was abolished after treatment with GW1100, a GPR40 antagonist (Figure 1B). This result indicates that HD6277‐induced Akt activation is not an off‐target effect and is relevant to GPR40 in C2C12 myoblasts. Treatment with HD6277 enhanced the levels of myogenic factors such as myocyte enhancer factor 2C (Mef2c), myogenin (MyoG) and MyHC. Elevation of these myogenic factors resulted in the promotion of myotube formation in C2C12 myoblasts (Figure S1A–C). These myogenic effects were also associated with the GPR40–Akt axis. The HD6277‐induced Mef2c transcript level decreased after treatment with an Akt inhibitor (Figure 1C). The expression of MyoG and MyHC that was accelerated by HD6277 was also suppressed by Akt inhibition (Figure 1D). Consequently, the inhibition of Akt phosphorylation reduced the MyHC‐positive area and myotube width in HD6277‐stimulated C2C12 myoblasts (Figure 1E). Furthermore, we found that the palmitate (200 μM)‐mediated reduction of Akt phosphorylation in C2C12 myoblasts recovered after HD6277 treatment (Figure 2A), and this effect disappeared after GW1100 treatment (Figure 2B). Under palmitate, the levels of myogenic factors and myotube formation were rescued after treatment with HD6277 (Figure S2A–C). These beneficial effects were regulated by Akt. Treatment with an Akt inhibitor attenuated HD6277‐induced transcription of Mef2c and myogenic factor 5 (Myf5), expressions of MyoG and MyHC and differentiation into myotubes in palmitate‐treated C2C12 myoblasts (Figure 2C–E). Our results suggest that HD6277 can induce myotube formation in an Akt‐dependent manner in C2C12 myoblasts with or without palmitate.

### 3.2 HD6277 Inhibited Palmitate‐Induced Cytotoxicity in an Akt‐Dependent Manner in C2C12 Myoblasts

We demonstrated that palmitate‐induced cytotoxic events in C2C12 myoblasts, which increased the B‐cell lymphoma 2 (Bcl‐2)–associated X protein (Bax)/Bcl‐2 ratio and cleaved caspase 3, were suppressed after treatment with HD6277 (Figure 3A). HD6277 also reversed the reduction of cell viabilities (Figure 3B) and enhancement of DNA fragmentation (Figure 3C) in palmitate‐treated C2C12 myoblasts. These HD6277‐mediated beneficial actions were all attenuated after treatment with an Akt inhibitor (Figure 3D–F). Our data demonstrate that the GPR40–Akt axis can inhibit palmitate‐induced cell damage in C2C12 myoblasts.

### 3.3 HD6277 Ameliorated H_2_O_2_‐Mediated Decrease in Myogenic Genes Expression and Increase in Cytotoxicity in C2C12 Myoblasts in an Akt‐Dependent Manner

It is well known that uncontrolled oxidative stress induces muscle damage [3]. Therefore, we investigated the ability of HD6277 to block H_2_O_2_‐induced harmful events in C2C12 myoblasts. The H_2_O_2_‐mediated reduction of Akt phosphorylation improved after treatment with HD6277, and this effect was cancelled by GW1100 (Figure S3A). Under H_2_O_2_ treatment, HD6277 increased the expression of the myogenic factors MyoG and MyHC, inhibited caspase 3 cleavage and improved cell viabilities (Figure S3B–D). These beneficial effects of HD6277 against H_2_O_2_ were all blocked by treatment with an Akt inhibitor (Figure S3E–G). We think that the GPR40–Akt axis can induce the expression of myogenic proteins and suppress cell damage in H_2_O_2_‐stimulated C2C12 myoblasts.

### 3.4 HD6277 Reduced Proteolytic Processes in an Akt‐Dependent Manner in Palmitate‐Treated C2C12 Myotubes

Unlike C2C12 myoblasts, 400‐μM palmitate was applied to C2C12 myotubes. This was because in C2C12 myotubes, the increase in atrogin‐1 and muscle RING‐finger protein 1 (MuRF1) expressions was not observed with treatment of 200‐μM palmitate but was seen with treatment of 400‐μM palmitate (data not shown). We found that in C2C12 myotubes, as in myoblasts, the palmitate‐mediated reduction of Akt phosphorylation was improved after HD6277 treatment (Figure 4A). This effect was blocked by GW1100 (Figure 4B), which suggests that Akt is a downstream molecule of muscle GPR40. HD6277 enhanced the phosphorylation of forkhead box protein O1A (FOXO1A), reduced the expression of E3 ubiquitin ligases atrogin‐1 and MuRF1 and inhibited protein ubiquitination, in palmitate‐treated C2C12 myotubes (Figure 4C,D). These HD6277‐mediated antiproteolytic effects were all reversed after treatment with an Akt inhibitor (Figure 4E,F). We additionally measured the global protein synthesis rate using puromycin. The palmitate‐induced decrease in protein synthesis was reversed after treatment with HD6277, and this effect was also abolished by an Akt inhibitor (Figure S4A,B). Our results show that the GPR40–Akt axis can reduce palmitate‐induced proteolytic events in C2C12 myotubes.

### 3.5 HD6277 Inhibited Cytotoxicity in an Akt‐Dependent Manner in Palmitate‐Treated C2C12 Myotubes

Consistent with the results in C2C12 myoblasts, HD6277 reduced the Bax/Bcl2 ratio and caspase 3 cleavage (Figure 5A), improved cell viabilities (Figure 5B) and suppressed DNA fragmentation (Figure 5C) in palmitate‐treated C2C12 myotubes. These benefits were all attenuated after treatment with an Akt inhibitor (Figure 5D–F). Our data suggest that the GPR40–Akt axis can inhibit palmitate‐induced cell damage in C2C12 myotubes.

### 3.6 HD6277 Inhibited the H_2_O_2_‐Mediated Increase in Proteolytic Processes and Cell Death in an Akt‐Dependent Manner in C2C12 Myotubes

In H_2_O_2_‐treated C2C12 myotubes, the level of Akt phosphorylation was increased after HD6277 treatment, and this elevation was blocked by GW1100 (Figure S5A). HD6277 improved the level of FOXO1A phosphorylation and reduced the expression of atrogin‐1 and MuRF1 in H_2_O_2_‐treated C2C12 myotubes (Figure S5B). The H_2_O_2_‐mediated increase in caspase 3 cleavage and decreased cell viability was reversed by HD6277 (Figure S5C,D). These HD6277‐mediated effects all disappeared after treatment with an Akt inhibitor (Figure S5E–G). Thus, we suggest that the GPR40–Akt axis can inhibit proteolytic events and cell death in H_2_O_2_‐stimulated C2C12 myotubes.

### 3.7 HD6277 Induced the Transcript Levels of Myogenic Genes and Inhibited the Expression of Proteolytic Molecules in Obese Mice

To identify the roles of GPR40 in the muscles of obese mice, C57BL/6 mice were fed a HFD or a HFD plus HD6277. HD6277 effectively lowered blood glucose but had no effect on lean mass, body weight or food intake (Figure S6A–E). Through Western blotting, we discovered that the levels of phosphorylated Akt in the muscles of HFD‐fed mice were reduced (Figure 6A). In contrast, the levels of phosphorylated Akt were significantly increased in the muscles of mice fed a HFD plus HD6277 (Figure 6A). However, the muscle GPR40 level did not changes in mice fed a HFD or a HFD plus HD6277 (Figure 6A). The levels of myogenic genes, such as paired box 3 (PAX3), PAX7, Mef2c, Myf5, myoblast determination protein 1 (MyoD) and MyoG, were all higher in the muscles from mice fed a HFD plus HD6277 than they were in those fed a HFD (Figure 6B). The HFD‐mediated reduction in FOXO1A phosphorylation, induced expression of atrogin‐1 and MuRF1, increased protein ubiquitination and enhanced Bax/Bcl2 ratio and caspase 3 cleavage were all reversed by the administration of HD6277 (Figure 6C,D). In addition, the decreased CSA of muscle fibres in HFD‐fed mice was significantly increased in HFD plus HD6277‐fed mice (Figure 6E). However, muscle strength was not affected by HD6277 (Figure 6F). These results show that GPR40 agonism induced the transcript levels of myogenic genes, inhibited the expression of E3 ubiquitin ligases and improved CSA but did not improve muscle strength in obese mice.

### 3.8 HD6277 Improved Muscle Strength via Inducing the Transcript Levels of Myogenic Genes and Reducing the Expression of Proteolytic Molecules in Aged Mice

We fed ND or ND plus HD6277 to aged mice to discover the roles of GPR40 agonism on age‐related muscle atrophy. Importantly, HD6277 administration increased lean mass and lowered blood glucose without changing body weight or food intake (Figure S7A–D). Western blotting showed that Akt phosphorylation was higher in the muscles of aged mice fed ND plus HD6277 than it was in the aged mice fed ND (Figure 7A). There was no difference in muscle GPR40 expression levels between aged mice fed ND and aged mice fed ND plus HD6277 (Figure 7A). The transcript levels of myogenic factors, such as PAX3, PAX7, Mef2c, Myf5, MyoD and MyoG, were all enhanced by HD6277 (Figure 7B). HD6277 increased FOXO1A phosphorylation, suppressed expression of atrogin‐1 and MuRF1, inhibited protein ubiquitination and reduced Bax/Bcl2 ratio and caspase 3 cleavage in muscles from aged mice (Figure 7C,D). Furthermore, the CSAs of muscle fibres and muscle strength were significantly increased by HD6277 treatment in aged mice (Figure 7E,F). We suggest that GPR40 agonism can ameliorate muscle atrophy and enhance muscle strength in aged mice.

## 4 Discussion

In this study, the following results were determined in GPR40 agonist‐treated C2C12 cells and muscle tissues from obese and aged mice. The GPR40 agonist, HD6277, (i) enhanced Akt phosphorylation and expression levels of myogenic factors, MyoG, Mef2c and MyHC; (ii) reduced palmitate‐mediated proteolytic events such as FOXO1 phosphorylation and E3 ubiquitin ligases expression; and (iii) improved palmitate‐induced cytotoxicity. (iv) These beneficial effects were all reversed by treatment with an Akt inhibitor. In addition, (v) HD6277 delayed the decline in muscle strength and muscle fibre thickness in aging mice but only improved muscle fibre thickness in obese mice.

Akt signalling plays a pivotal role in regulating hypertrophy and atrophy of skeletal muscle. Bodine et al. reported that Akt phosphorylation increases during muscle hypertrophy and decreases during muscle atrophy; they also found that genetic activation of Akt signalling can increase myofiber size [18]. In C2C12 myoblasts, the inhibition of Akt signalling limits MyoG expression and cell motility, which leads to reduced MyHC levels and myotube formation [19]. Skeletal muscle‐specific Akt deficiency results in debilitation, osteopenia and shortened lifespan as well as muscle atrophy in mice [20]. Furthermore, some study has found that GPR40 can regulate Akt signalling. In renal carcinoma cells (RCCs), oleic acid, an FFA, enhanced Akt phosphorylation via the GPR40/integrin‐linked kinase (ILK) axis [21]. Karmokar and Moniri reported that treatment with AS2034178, a selective GPR40 agonist, can modulate cell proliferation in an Akt‐dependent manner in RCCs [22]. Based on these reports, we focused initially on whether muscular Akt signalling is regulated by GPR40 agonism. In C2C12 myoblasts, HD6277 increased Akt phosphorylation, which was reversed by the GPR40 antagonist, GW1100 (Figure 1A,B). Palmitate‐mediated reduction of Akt phosphorylation was cancelled after treatment with HD6277 in C2C12 myoblast and myotubes (Figures 2A,B and 4A,B). Furthermore, attenuated Akt signalling in muscle tissues from obese and aged mice recovered after HD6277 administration (Figures 6A and 7A). Unlike in RCC [21], GPR40‐mediated muscular Akt phosphorylation was not modulated by ILK (data not shown). Therefore, in the present study, we confirmed the benefits of muscle GPR40 via Akt signalling.

We determined whether the expression of several myogenic factors was regulated by the GPR40–Akt axis. In C2C12 myoblasts, HD6277 induced the expression of myogenic factors, MyoG, Mef2c and MyHC, and myotube formation, and these effects were all attenuated after the administration of an Akt inhibitor (Figure 1C–E). On palmitate treatment, reduced myogenic events were restored by HD6277 in an Akt‐dependent manner (Figure 2C–E). In addition, muscular phosphorylated Akt, PAX3, PAX7, Mef2c, Myf5, MyoD and MyoG levels were all increased after HD6277 administration in obese and aged mice (Figures 6B and 7B). Muscle regeneration is a cellular response regulated by a variety of molecules to repair muscle injury [6]. When muscles are damaged, satellite cells are activated and differentiate into myoblasts, which turn into myocytes and then fuse to form muscle fibres [23]. Myf5 regulates the activation of satellite cells and mediates the expression of MyoG and Mef2, which enhance myotube formation [24, 25]. PAX3 and PAX7 are markers for primitive myogenic cell populations [26]. The levels of these myogenic factors are lower in elderly individuals with sarcopenia than they are in those without sarcopenia [27]. Brzeszczyńska et al. cultured primary skeletal muscle cells from young and old subjects and found that the aged cells had lower expression of the myogenic factors MyoG and MyoD and higher expression of pro‐inflammatory cytokines than did the young cells during myogenesis [27]. Enhanced expression and stabilization of MyoG were strongly associated with an increase in MyHC expression and muscle fibre diameter in dexamethasone‐treated mice [28]. In addition, impaired myogenesis can also be induced by obese subcutaneous adipose tissue, contributing to reduced myotube thickness, particularly in old skeletal muscle [29]. These reports support our results that the GPR40–Akt axis can inhibit muscle atrophy by enhancing myogenic factors.

Muscle atrophy can also be accelerated by proteolysis. Atrogin‐1 and MuRF1, known as E3 ubiquitin ligases, promote muscular protein degradation, leading to a reduction in muscle fibre size [30]. These ligases are expressed more with age in humans and rodents [31, 32]. Castillero et al. showed that the suppression of atrogin‐1 and MuRF1 restored dexamethasone‐mediated decreased myotube diameter by inhibiting protein degradation and enhancing protein synthesis [33]. These ligases can be regulated by Akt–FOXO signalling. FOXO, a transcription factor for atrogin‐1 and MuRF, moves from the nucleus to the cytoplasm after it is phosphorylated by Akt; this movement indicates that phosphorylated FOXO is inactive [30]. Therefore, FOXO‐deficient muscles had lower E3 ligase levels and larger fibre CSA than normal muscles [34]. In the present study, we show that HD6277 reversed the palmitate‐mediated decreases in FOXO1A phosphorylation, and this effect was cancelled out by Akt inhibitor in C2C12 myotubes (Figure 4C,D). Palmitate‐induced the levels of atrogin‐1, MuRF1 and protein ubiquitination; these changes all decreased after treatment with HD6277 in an Akt‐dependent manner (Figure 4C,D). Similar to the in vitro study, HD6277 administration increased Akt and FOXO1 phosphorylation and decreased the levels of atrogin‐1, MuRF1 and protein ubiquitination in obese and aged mice (Figures 6C and 7C). Furthermore, HD6277 enhanced lean mass, reduced fat mass and restored a decrease in muscle strength and myofiber CSA in aging mice (Figures S4A and S7E,F) but only improved myofiber CSA in obese mice (Figures S3A and S6E,F). Similar to our data, treatment with GW1100, a GPR40 antagonist, reduced the myofiber CSA in lung carcinoma‐bearing mice [35]. Therefore, we suggest that the GPR40–Akt axis may be a novel modulator that prevents muscle protein degradation.

Importantly, we also reported that HD6277 inhibited muscular cytotoxicity. Muscle apoptosis increases with age or obesity and leads to a reduction in the satellite cell population, decreased capacity for muscle regeneration and increased proteolysis [3, 36]. In C2C12 myoblasts and myotubes, palmitate‐mediated apoptotic events, caspase 3 cleavage and DNA fragmentation were attenuated after treatment with HD6277 in an Akt‐dependent mechanism (Figures 3 and 5). These HD6277‐mediated antiapoptotic effects were accompanied by increased myogenic factors and reduced ubiquitin–proteasome activity in the muscles from obese and aged mice (Figures 6 and 7). Consistent with our data, GPR40‐specific small chemicals triggered anticytotoxicity effects in various cell types. In pancreatic beta cells, CNX‐011‐67, a GPR40 agonist, inhibited ROS production and caspase 3 activity, resulting in increased cell viability under inflammatory stimuli [12]. In vascular endothelial cells, treatment with AM1638, another GPR40 agonist, reduced the palmitate‐induced DNA condensation and fragmentation [16]. Sun et al. reported that ultraviolet B (UV‐B)–induced oxidative stress and the release of lactate dehydrogenase (LDH) were associated with a decrease in GPR40 expression; these cytotoxic effects were blocked by GPR40 agonism in epidermal stem cells [37]. Based on these results, we believe that GPR40 agonism has a protective role against cytotoxicity in muscles and other cell types.

Our results indicate that HD6277 promotes myogenic factors and reduces protein degradation in both obese and aging models. However, improvements in four‐limb muscle strength (Figures 6F and 7F) and body muscle percentage (Figures S6A and S7A) were observed only in the aging model. To elucidate this difference, we further investigated other factors contributing to muscle atrophy and identified differences in mitochondrial function between the both models. Mitochondrial oxidative phosphorylation (OXPHOS) proteins, mitochondrial content and ATP production were all higher in aged mice fed a ND plus HD6277 than they were in aged mice fed a ND alone (Figure S8A–C). However, the HFD‐induced reduction in mitochondrial performance was not rescued by HD6277 administration (Figure S9A–C). Mitochondrial dysfunction is believed to be a key cause of sarcopenia, along with declined muscle regeneration and enhanced proteolysis [38]. In humans, muscle mitochondrial content and the ATP production rate decrease with advancing age, which is strongly linked to increased oxidative damage during aging [39]. Fujii et al. reported that treatment with 5‐aminolevulinic acid, an element of mitochondrial electron carriers, improved muscle mass and performance by restoring mitochondrial quantity in aged mice [40]. Our results show that GPR40 agonism improves muscle performance by regulating mitochondrial function in aged mice.

However, a limitation of our study is the lack of a direct assessment of autophagic flux. Given that autophagy, along with E3 ubiquitin ligases and mitochondria, plays a crucial role in muscle atrophy, further research is needed to elucidate how GPR40 influences autophagic flux in muscle tissue [3, 6]. Despite this limitation, our findings provide valuable insights into the role of GPR40 in muscle atrophy. Future studies exploring the relationship between GPR40 activation, autophagic flux and other regulatory factors involved in muscle degradation are necessary to better understand the contribution of GPR40 to sarcopenia.

**FIGURE 8:**
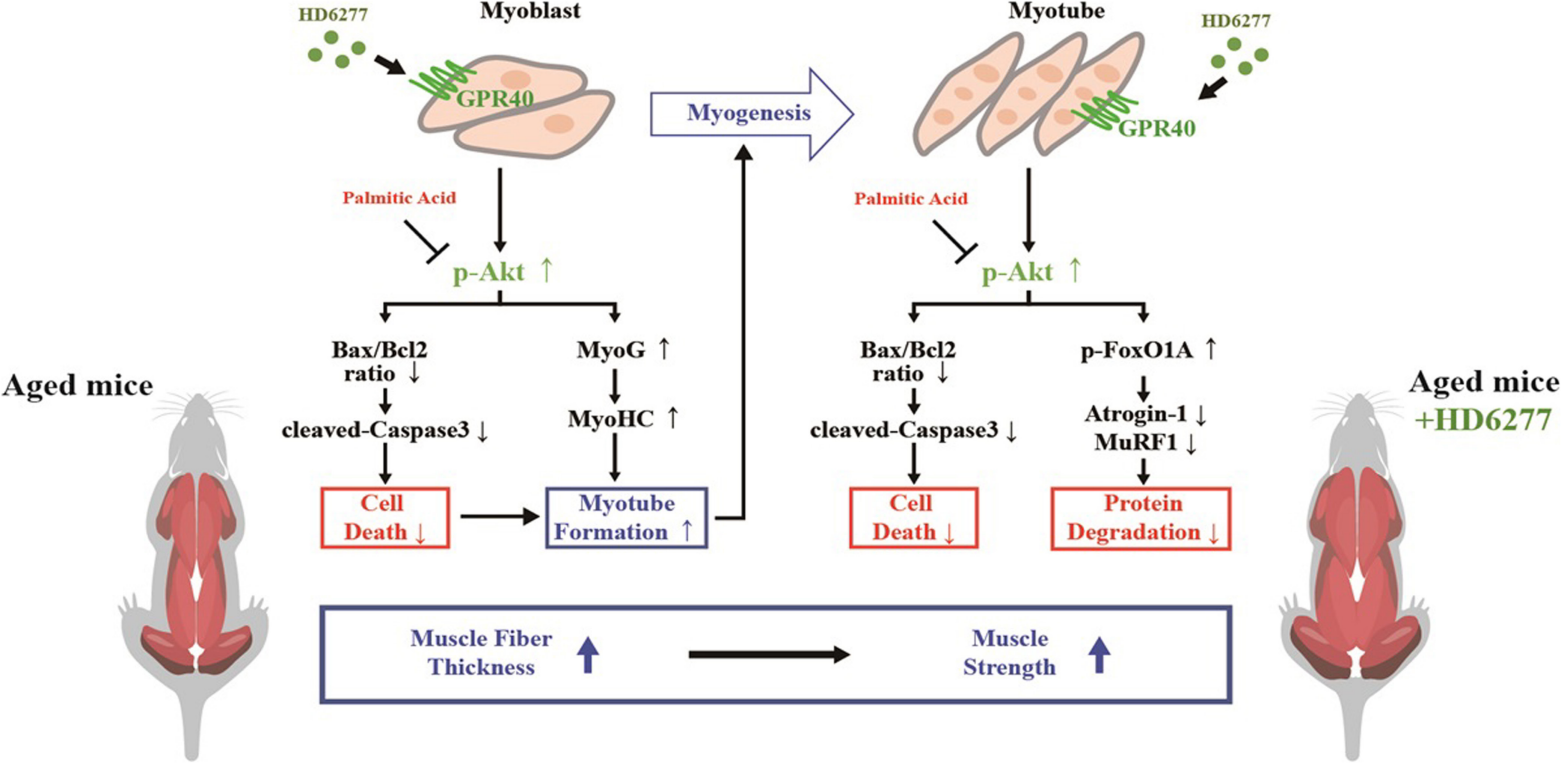
Schematic diagram of HD6277 functions in muscles. The HD6277‐Akt axis enhanced myogenic factors, reduced proteolytic processes and inhibited cytotoxicity in C2C12 cells and muscle tissues from obese and aged mice. Furthermore, HD6277 administration improved muscle strength and the CSA of muscle fibres in aged mice.

In conclusion, we have revealed that HD6277, a small chemical for GPR40 agonism, increases myogenic factors, reduces the ubiquitin–proteasome system and inhibits cytotoxicity; through these effects, HD6277 improves muscle strength and myofiber CSA in aged mice (Figure 8). These results suggest that GPR40 could become a therapeutic target to overcome sarcopenia.

## Disclosure

The authors have nothing to report.

## Conflicts of Interest

The authors declare no conflicts of interest.

## Supporting information


**Figure S1.** HD6277 enhanced the myogenic processes in C2C12 myoblasts. (A) The cells were differentiated with various doses of HD6277 (0, 10 or 25 μM) for 2 days, and then, the transcript levels of Mef2c and Myf5 were analysed by qPCR. (B) Western blotting was performed to determine the levels of MyoG and MyHC. (C) MyHC‐stained cells (green) were observed under a fluorescence microscope, and MyHC‐positive area and myotube width were calculated using the ImageJ program. Scale bar: 275 μm. HD, HD6277. Error bars indicate the mean ± SD (*p < 0.05; ANOVA with post hoc *t* test).
**Figure S2.** HD6277 reversed the palmitate‐mediated reduction in the myogenic processes in C2C12 myoblasts. (A) The cells were stimulated with palmitate (200 μM) alone or together with HD6277 (10 or 25 μM) and then differentiated for 2 days. The transcript levels of Mef2c and Myf5 were determined by qPCR. (B) Western blotting was performed to confirm the levels of MyoG and MyHC. (C) MyHC‐positive area and myotube width were calculated in images stained with MyHC (green fluorescence). Scale bar: 275 μm. HD, HD6277; PA, palmitate; Veh, vehicle. Error bars indicate the mean ± SD (*p < 0.05; ANOVA with post hoc *t* test).
**Figure S3.** HD6277 prevented the H_2_O_2_‐mediated harmful effects via an Akt‐dependent manner in C2C12 myoblasts. (A) The cells were stimulated with H_2_O_2_ (100 μM), H_2_O_2_ plus HD6277 (25 μM) or H_2_O_2_ plus HD6277 with GW1100 (10 μM). The phosphorylated Akt level was analysed by Western blotting. (B,E) The cells were stimulated with H_2_O_2_ (100 μM), H_2_O_2_ plus HD6277 (10 or 25 μM) or H_2_O_2_ plus HD6277 with Akt inhibitor (2 μM) and then differentiated for 2 days. MyoG and MyHC levels were analysed by Western blotting. (C,F) The cells were stimulated with H_2_O_2_ (100 μM), H_2_O_2_ plus HD6277 (25 μM) or H_2_O_2_ plus HD6277 with Akt inhibitor (2 μM), and then, the cleaved caspase 3 level was determined by Western blotting. (D,G) Cell viability was measured using EZ‐Cytox solution. Akti, Akt inhibitor; GW, GW1100; HD, HD6277; Veh, vehicle. Error bars indicate the mean ± SD (*p < 0.05; ANOVA with post hoc *t* test).
**Figure S4.** HD6277 prevented the palmitate‐mediated decrease in protein synthesis via an Akt‐dependent manner in C2C12 myotubes. (A,B) Fully differentiated cells were incubated with palmitate (400 μM), palmitate plus HD6277 (10 or 25 μM) or palmitate plus HD6277 with Akt inhibitor (2 μM) and then stimulated with puromycin (10 μg/mL) for 1 h. Puromycin‐tagged proteins were detected using Western blotting, and CBB staining was performed as a loading control. Akti, Akt inhibitor; CBB, Coomassie brilliant blue; HD, HD6277; PA, palmitate; Veh, vehicle. Error bars indicate the mean ± SD (*p < 0.05; ANOVA with post hoc *t* test).
**Figure S5.** The HD6277‐Akt axis inhibited H_2_O_2_‐mediated proteolysis and cell death in C2C12 myotubes. (A) The cells were incubated with H_2_O_2_ (100 μM), H_2_O_2_ plus HD6277 (25 μM) or H_2_O_2_ plus HD6277 with GW1100 (10 μM). The phosphorylated Akt level was detected by Western blotting. (B,E) The cells were stimulated with H_2_O_2_ (100 μM), H_2_O_2_ plus HD6277 (10 or 25 μM) or H_2_O_2_ plus HD6277 with Akt inhibitor (2 μM). The phosphorylated FOXO1A, atrogin‐1 and MuRF1 levels were determined by Western blotting. (C,F) The cleaved caspase 3 level was detected by Western blotting. (D,G) Cell viability was measured using EZ‐Cytox solution. Akti, Akt inhibitor; GW, GW1100; HD, HD6277; Veh, vehicle. Error bars indicate the mean ± SD (*p < 0.05; ANOVA with post hoc *t* test).
**Figure S6.** Mice were fed a high‐fat diet mixed with or without HD6277 for 10 weeks. (A) Fat mass and lean mass were analysed using an animal DEXA scanner. Red dots indicate lipids. (B) Body weight and (C) food intake were measured weekly for 10 weeks. (D) IPGTT and (E) IPITT were performed 2 weeks before sacrifice. HFD, high‐fat diet–fed mice; HFD + HD, HFD mixed with HD6277‐fed mice; ND, normal diet–fed mice. Error bars indicate the mean ± SD (*p < 0.05; ANOVA with post hoc *t* test).
**Figure S7.** Aged mice were fed a normal diet with or without HD6277 for 22 weeks. (A) Changes in body composition were identified using an animal DEXA scanner. Red dots indicate lipids. (B) Body weight and (C) food intake were recorded weekly for 22 weeks. (D) IPGTT was performed 2 weeks before sacrifice. Aged, normal diet–fed aged mice; Aged + HD, normal diet mixed with HD6277‐fed aged mice. Error bars indicate the mean ± SD (*p < 0.05; two‐tailed *t* tests assuming unequal variance).
**Figure S8.** HD6277 administration improved muscle mitochondrial performance in aged mice. (A) Mitochondrial oxidative phosphorylation (OXPHOS) proteins were detected by Western blotting. (B) Mitochondrial contents were calculated by qPCR. (C) ATP production rates were measured using a commercial ATP assay kit. Aged, normal diet–fed aged mice; Aged + HD, normal diet mixed with HD6277‐fed aged mice. Error bars indicate the mean ± SD (*p < 0.05; two‐tailed *t* tests assuming unequal variance).
**Figure S9.** HD6277 administration did not affect muscle mitochondrial performance in obese mice. (A) Mitochondrial OXPHOS proteins were determined by Western blotting. (B) Mitochondrial contents were measured by qPCR. (C) ATP production was calculated using a commercial ATP assay kit. HFD, high‐fat diet–fed mice; HFD + HD, HFD mixed with HD6277‐fed mice; ND, normal diet–fed mice; *ND*, no detect; *ns*, no significant. Error bars indicate the mean ± SD (ANOVA with post hoc *t* test).
**Table S1.** Antibody Lists used in the present study.
**Table S2.** Primer sets used in the present study.
