# Supplementary material for: HD6277 Suppresses Muscle Atrophy by Promoting Myogenic Factors and Inhibiting Proteolysis in Aged Mice
Source: J Cachexia Sarcopenia Muscle. 2025 Apr 14;16(2):e13805. doi: 10.1002/jcsm.13805 (PMC11996700; doi:10.1002/jcsm.13805)
Supplement: Supplementary file 1 — Figure S1. HD6277 enhanced the myogenic processes in C2C12 myoblasts. (A) The cells were differentiated with various doses of HD6277 (0, 10 or 25 μM) for 2 days, and then, the transcript levels of Mef2c and Myf5 were analysed by qPCR. (B) Western blotting was performed to determine the levels of MyoG and MyHC. (C) MyHC‐stained cells (green) were observed under a fluorescence microscope, and MyHC‐positive area and myotube width were calculated using the ImageJ program. Scale bar: 275 μm. HD, HD6277. Error bars indicate the mean ± SD (*p < 0.05; ANOVA with post hoc t test). Figure S2. HD6277 reversed the palmitate‐mediated reduction in the myogenic processes in C2C12 myoblasts. (A) The cells were stimulated with palmitate (200 μM) alone or together with HD6277 (10 or 25 μM) and then differentiated for 2 days. The transcript levels of Mef2c and Myf5 were determined by qPCR. (B) Western blotting was performed to confirm the levels of MyoG and MyHC. (C) MyHC‐positive area and myotube width were calculated in images stained with MyHC (green fluorescence). Scale bar: 275 μm. HD, HD6277; PA, palmitate; Veh, vehicle. Error bars indicate the mean ± SD (*p < 0.05; ANOVA with post hoc t test). Figure S3. HD6277 prevented the H2O2‐mediated harmful effects via an Akt‐dependent manner in C2C12 myoblasts. (A) The cells were stimulated with H2O2 (100 μM), H2O2 plus HD6277 (25 μM) or H2O2 plus HD6277 with GW1100 (10 μM). The phosphorylated Akt level was analysed by Western blotting. (B,E) The cells were stimulated with H2O2 (100 μM), H2O2 plus HD6277 (10 or 25 μM) or H2O2 plus HD6277 with Akt inhibitor (2 μM) and then differentiated for 2 days. MyoG and MyHC levels were analysed by Western blotting. (C,F) The cells were stimulated with H2O2 (100 μM), H2O2 plus HD6277 (25 μM) or H2O2 plus HD6277 with Akt inhibitor (2 μM), and then, the cleaved caspase 3 level was determined by Western blotting. (D,G) Cell viability was measured using EZ‐Cytox solution. Akti, Akt inhibitor; GW, GW1100; [file JCSM-16-e13805-s001.docx]

**Supplementary Data**

**Supplementary Methods**

**The Treatment of Hydroperoxide (H_2_O_2_)**

We treated C2C12 cells with H_2_O_2_ (Sigma-Aldrich, MO, USA) instead of palmitate because oxidative stress is believed to be a major cause of muscle atrophy along with lipotoxicity.^1^ We evaluated the roles of HD6277 in C2C12 myoblasts and myotubes stimulated with 100 µM H_2_O_2_.

**The evaluation of the protein synthesis rate**

To measure the protein synthesis rate, C2C12 myotubes were incubated with puromycin (Merck Millipore, MA, USA) for 1 h, and puromycin-tagged proteins were detected by Western blotting using a puromycin antibody (Abcam, Cambridge, UK). Puromycin can interact with tRNA and become anchored to a protein that is being synthesized. ^2^

**The measurement of body composition**

Dual energy X-ray absorptiometry scan (DEXA) (InAlyzer, Medikors, Sungnam, Republic of Korea) was used to measure changes in the body composition of the animals according to the instruction manual.

**Intraperitoneal Glucose Tolerance Tests (IPGTT) and Intraperitoneal Insulin Tolerance Test (IPITT)**

All animals underwent IPGTT and IPITT two weeks before they were sacrificed. After fasting for 6 hours, basal blood glucose levels (0 min) were measured, followed by an intraperitoneal injection of glucose (1 g per kg) or insulin (0.5 Unit/kg). Blood samples were collected from the tail vein at specified intervals, and blood glucose levels were measured using a glucometer (Accu-Check Performa, Roche, Swiss) according to the user guideline.

**The calculation of mitochondrial contents**

Muscle deoxyribonucleic acid (DNA) was isolated using lysis buffer (consisting of 100 mM Tris-HCl; pH 8.5, 5 mM EDTA, 0.2% SDS, 200 mM NaCl, 100 ug proteinase K/ml) and used for quantitative real-time PCR. Mitochondrial contents were calculated using the ratio of NADH dehydrogenase subunit I (Nd1), a mitochondrial gene, to lipoprotein lipase (Lpl), a genomic gene. ^3^

**The analysis of adenosine triphosphate (ATP) production levels**

Muscle ATP levels were measured using an EZ-ATP Assay Kit (Dogen Bio, Seoul, South Korea) according to the user manual.

**Supplementary Figures**

**
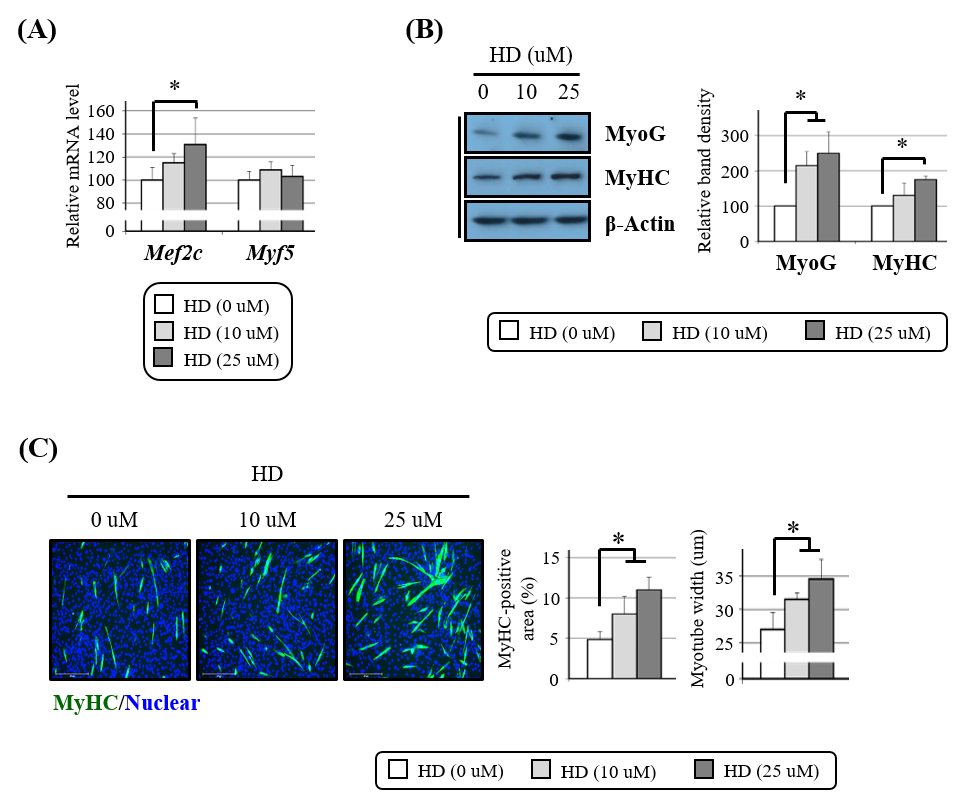
**

**Supplementary Figure 1.** HD6277 enhanced the myogenic processes in C2C12 myoblasts. (A) The cells were differentiated with various doses of HD6277 (0, 10, or 25 μM) for 2 days, and then the transcript levels of Mef2c and Myf5 were analyzed by qPCR. (B) Western blotting was performed to determine the levels of MyoG and MyHC. (C) MyHC-stained cells (green) were observed under a fluorescence microscope, and MyHC-positive area and myotube width were calculated using the ImageJ program. Scale bar; 275 μm. *HD*; HD6277. Error bars indicate the mean ± SD (*, *P* <0.05; ANOVA with post hoc *t*-test).


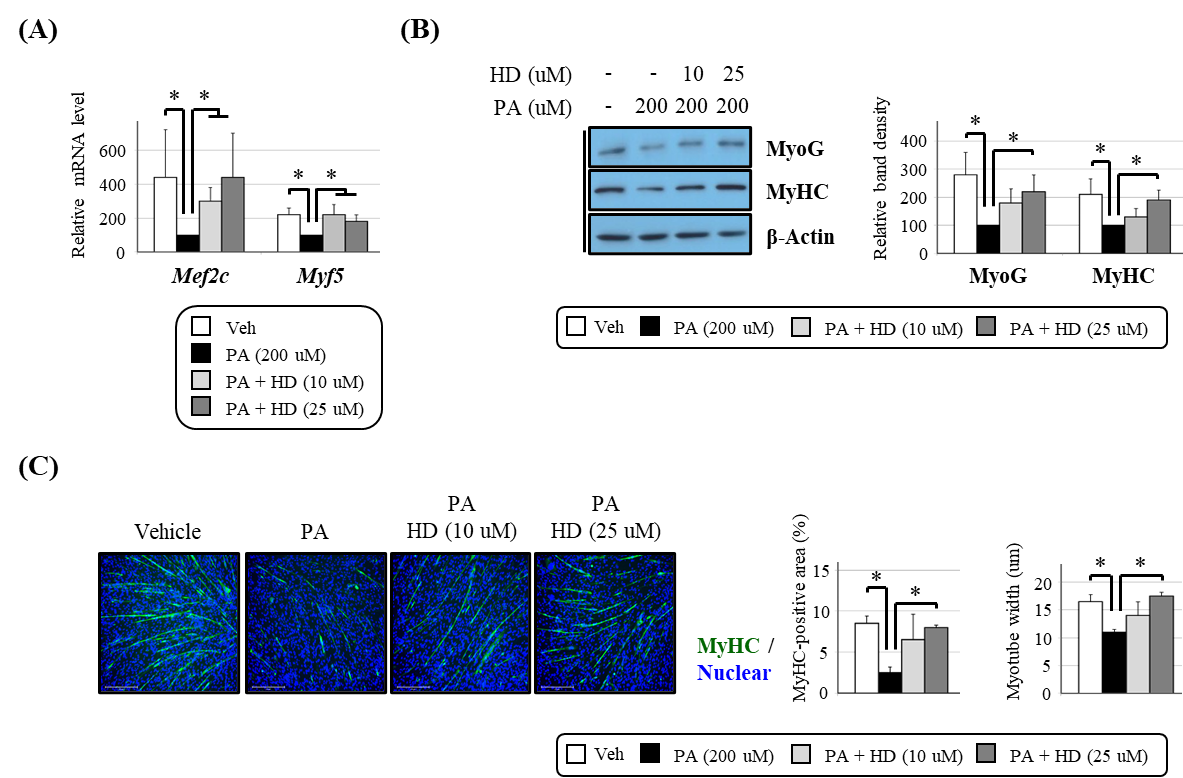


**Supplementary Figure 2.** HD6277 reversed the palmitate-mediated reduction in the myogenic processes in C2C12 myoblasts. (A) The cells were stimulated with palmitate (200 μM) alone or together with HD6277 (10 or 25 μM), and then differentiated for 2 days. The transcript levels of Mef2c and Myf5 were determined by qPCR. (B) Western blotting was performed to confirm the levels of MyoG and MyHC. (C) MyHC-positive area and myotube width were calculated in images stained with MyHC (green fluorescence). Scale bar; 275 μm. *Veh*; vehicle, *PA*; palmitate, *HD*; HD6277. Error bars indicate the mean ± SD (*, *P* <0.05; ANOVA with post hoc *t*-test).


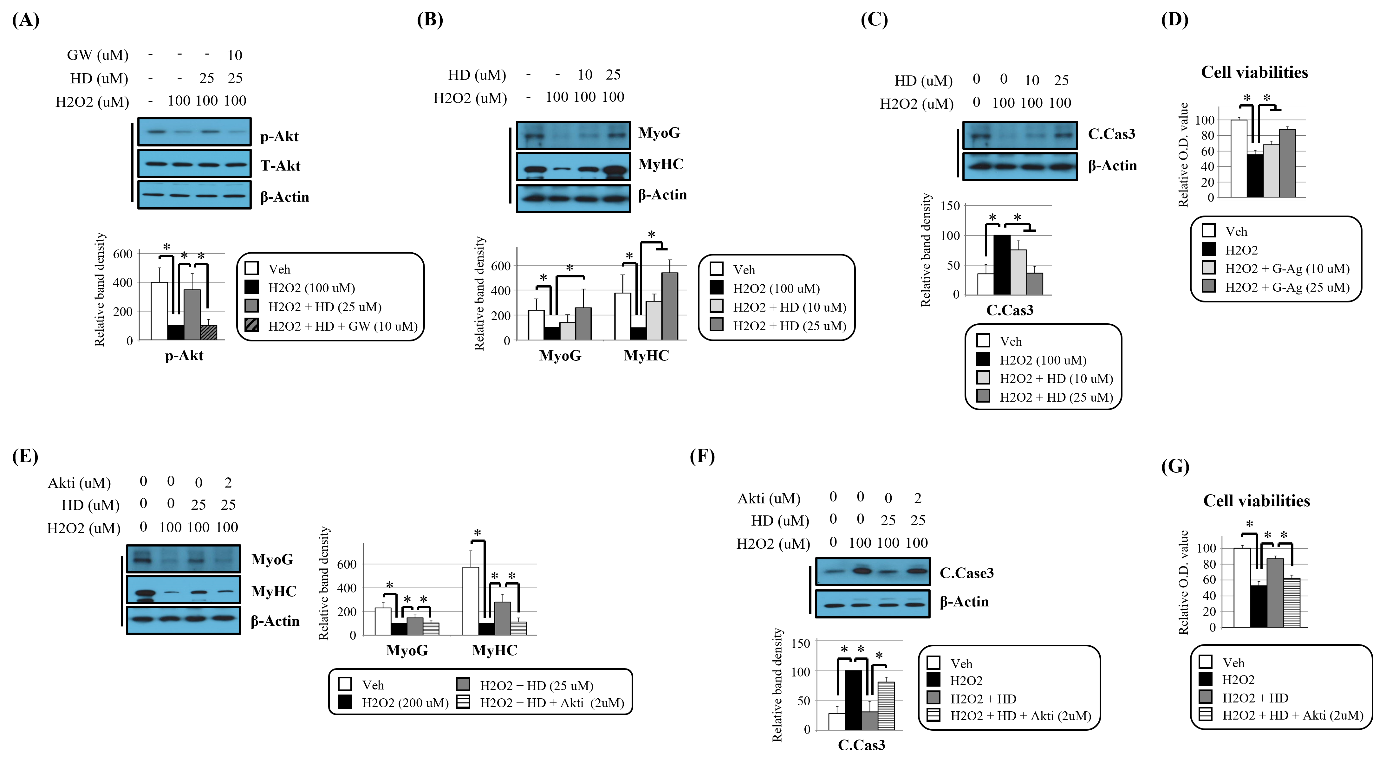


**Supplementary Figure 3.** HD6277 prevented the H_2_O_2_-mediated harmful effects via an Akt dependent manner in C2C12 myoblasts. (A) The cells were stimulated with H_2_O_2_ (100 μM), H_2_O_2_ plus HD6277 (25 μM), or H_2_O_2_ plus HD6277 with GW1100 (10 μM). The phosphorylated Akt level was analyzed by Western blotting. (B, E) The cells were stimulated with H_2_O_2_ (100 μM), H_2_O_2_ plus HD6277 (10 or 25 μM), or H_2_O_2_ plus HD6277 with Akt inhibitor (2 μM), and then differentiated for 2 days. MyoG and MyHC levels were analyzed by Western blotting. (C, F) The cells were stimulated with H_2_O_2_ (100 μM), H_2_O_2_ plus HD6277 (25 μM), or H_2_O_2_ plus HD6277 with Akt inhibitor (2 μM), and then the cleaved caspase 3 level was determined by Western blotting. (D, G) Cell viability was measured using EZ-Cytox solution. *Veh*, vehicle; *HD*, HD6277; *GW,* GW1100; *Akti*, Akt inhibitor. Error bars indicate the mean ± SD (*, *P* <0.05; ANOVA with post hoc *t*-test).


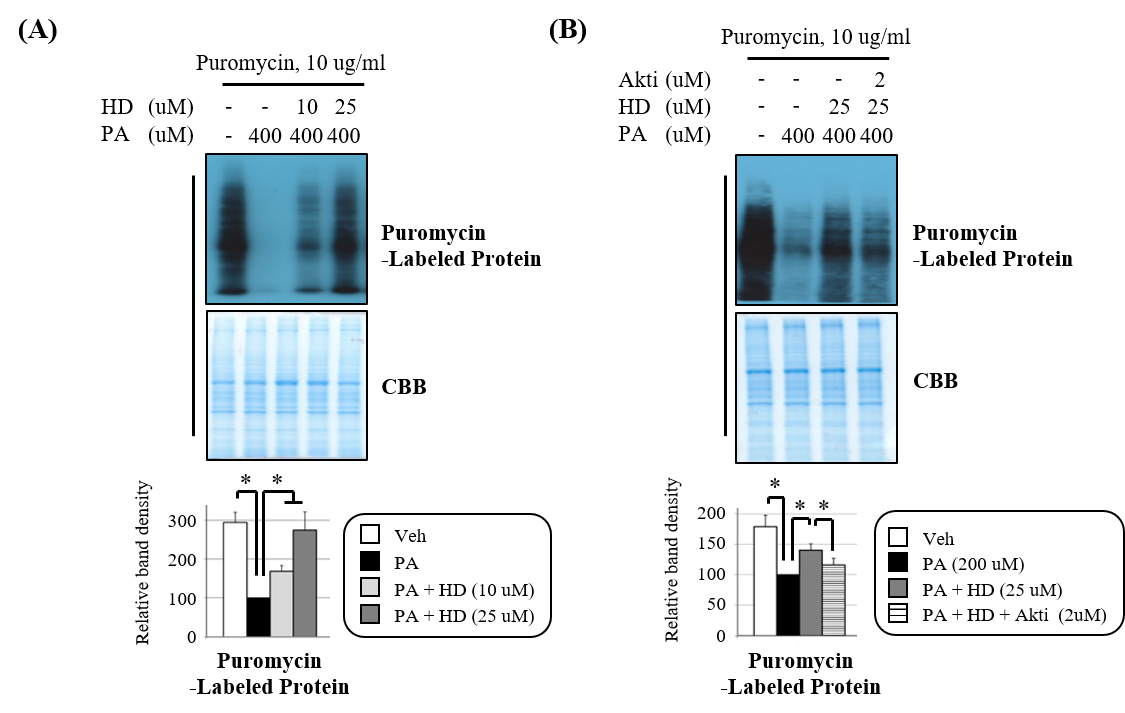


**Supplementary Figure 4.** HD6277 prevented the palmitate-mediated decrease in protein synthesis via an Akt dependent manner in C2C12 myotubes. (A, B) Fully differentiated cells were incubated with palmitate (400 μM), palmitate plus HD6277 (10 or 25 μM), or palmitate plus HD6277 with Akt inhibitor (2 μM), and then stimulated with puromycin (10 μg/ml) for 1 h. Puromycin-tagged proteins were detected using Western blotting, and CBB staining was performed as a loading control. *Veh*, vehicle; *PA*, palmitate; *HD*, HD6277; *Akti*, Akt inhibitor; CBB, coomassie brilliant blue. Error bars indicate the mean ± SD (*, *P* <0.05; ANOVA with post hoc *t*-test).


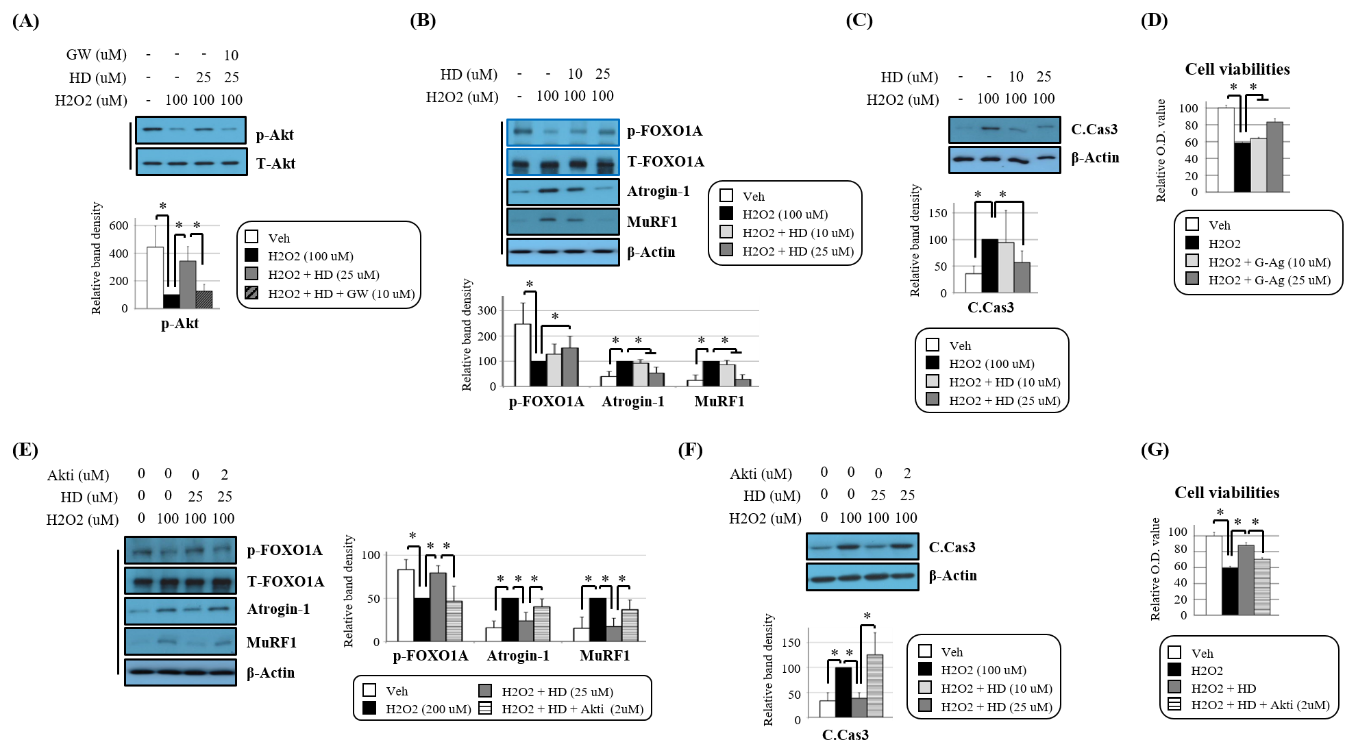


**Supplementary Figure 5.** The HD6277-Akt axis inhibited H_2_O_2_-mediated proteolysis and cell death in C2C12 myotubes. (A) The cells were incubated with H_2_O_2_ (100 μM), H_2_O_2_ plus HD6277 (25 μM), or H_2_O_2_ plus HD6277 with GW1100 (10 μM). The phosphorylated Akt level was detected by Western blotting. (B, E) The cells were stimulated with H_2_O_2_ (100 μM), H_2_O_2_ plus HD6277 (10 or 25 μM), or H_2_O_2_ plus HD6277 with Akt inhibitor (2 μM). The phosphorylated FOXO1A, atrogin-1, and MuRF1 levels were determined by Western blotting. (C, F) The cleaved caspase 3 level was detected by Western blotting. (D, G) Cell viability was measured using EZ-Cytox solution. *Veh*, vehicle; *HD*, HD6277; *GW,* GW1100; *Akti*, Akt inhibitor. Error bars indicate the mean ± SD (*, *P* <0.05; ANOVA with post hoc *t*-test).


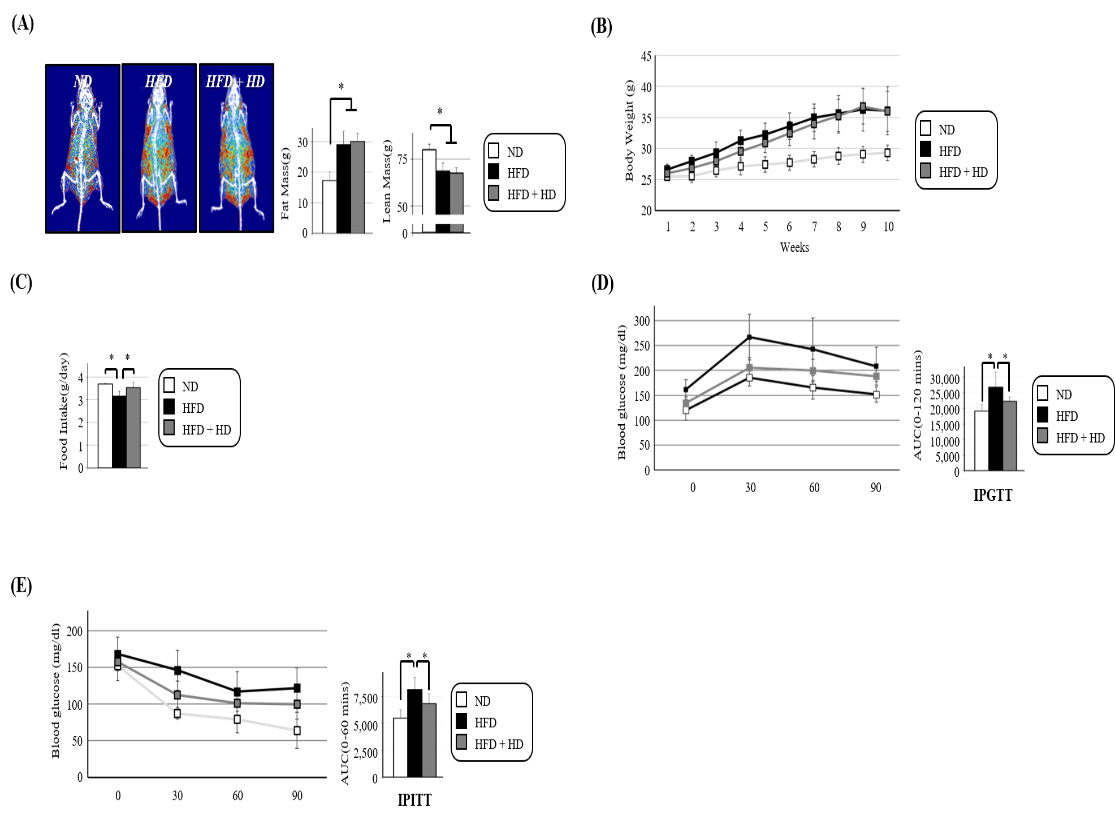


**Supplementary Figure 6.** Mice were fed a high-fat diet mixed with or without HD6277 for 10 weeks. (A) Fat mass and lean mass were analyzed using an animal DEXA scanner. Red dots indicate lipids. (B) Body weight and (C) food intake were measured weekly for 10 weeks. (D) IPGTT and (E) IPITT were performed two weeks before sacrifice. *ND*, normal diet-fed mice; *HFD*, high-fat diet-fed mice; *HFD+HD*, HFD mixed with HD6277-fed mice. Error bars indicate the mean ± SD (*, *P* <0.05; ANOVA with post hoc *t*-test).


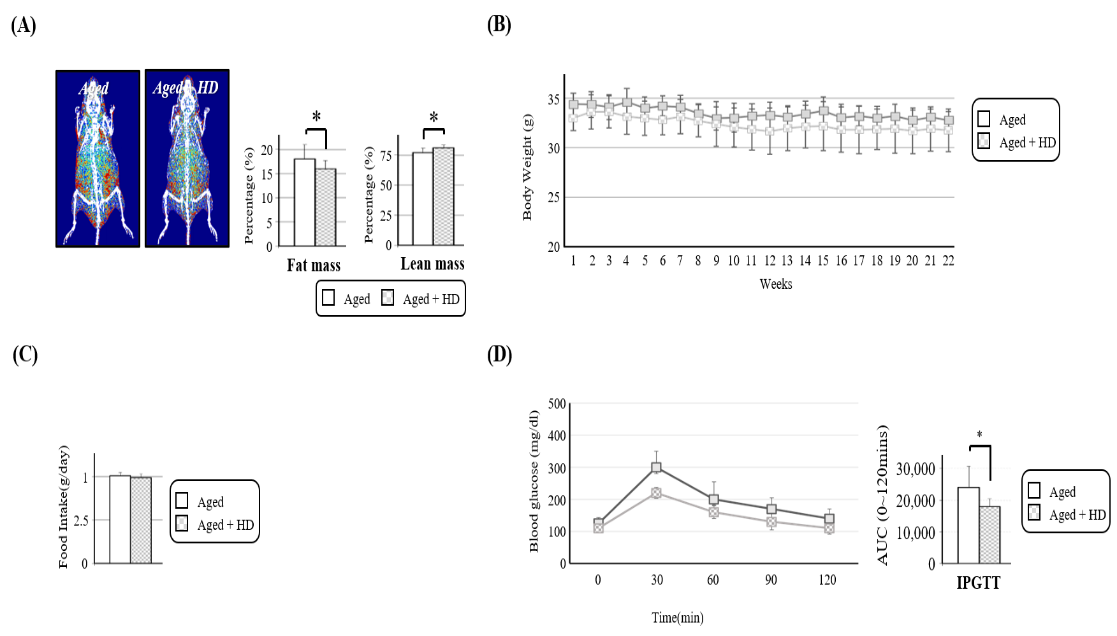


**Supplementary Figure 7.** Aged mice were fed a normal diet with or without HD6277 for 22 weeks. (A) Changes in body composition were identified using an animal DEXA scanner. Red dots indicate lipids. (B) Body weight and (C) food intake were recorded weekly for 22 weeks. (D) IPGTT was performed two weeks before sacrifice. *Aged*, normal diet-fed aged mice; *Aged+HD*, normal diet mixed with HD6277-fed aged mice. Error bars indicate the mean ± SD (*, *P* <0.05; two-tailed *t*-tests assuming unequal variance).


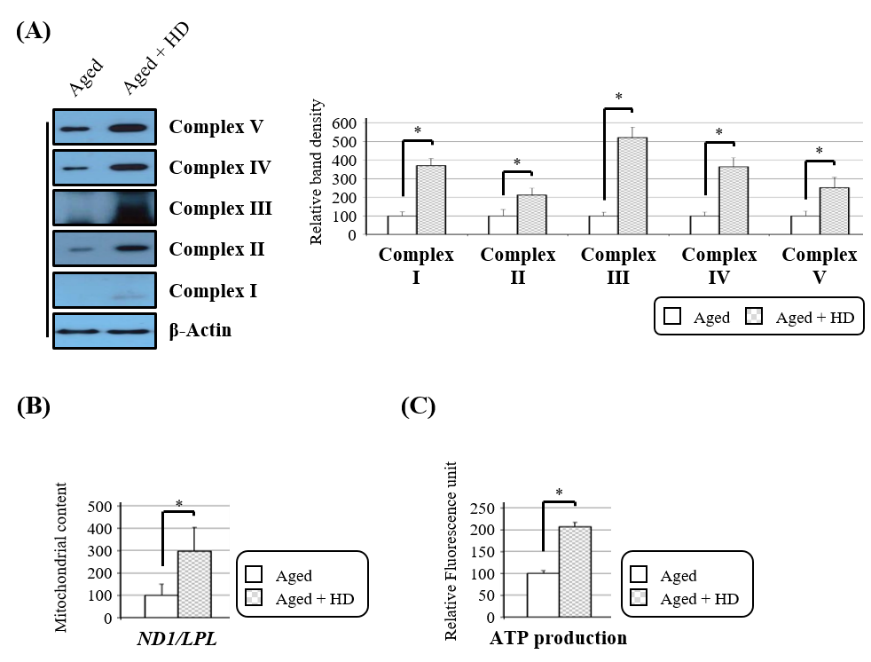


**Supplementary Figure 8.** HD6277 administration improved muscle mitochondrial performance in aged mice. (A) Mitochondrial oxidative phosphorylation (OXPHOS) proteins were detected by Western blotting. (B) Mitochondrial contents were calculated by qPCR. (C) ATP production rates were measured using a commercial ATP assay kit. *Aged*, normal diet-fed aged mice; *Aged+HD*, normal diet mixed with HD6277-fed aged mice. Error bars indicate the mean ± SD (*, *P* <0.05; two-tailed *t*-tests assuming unequal variance).


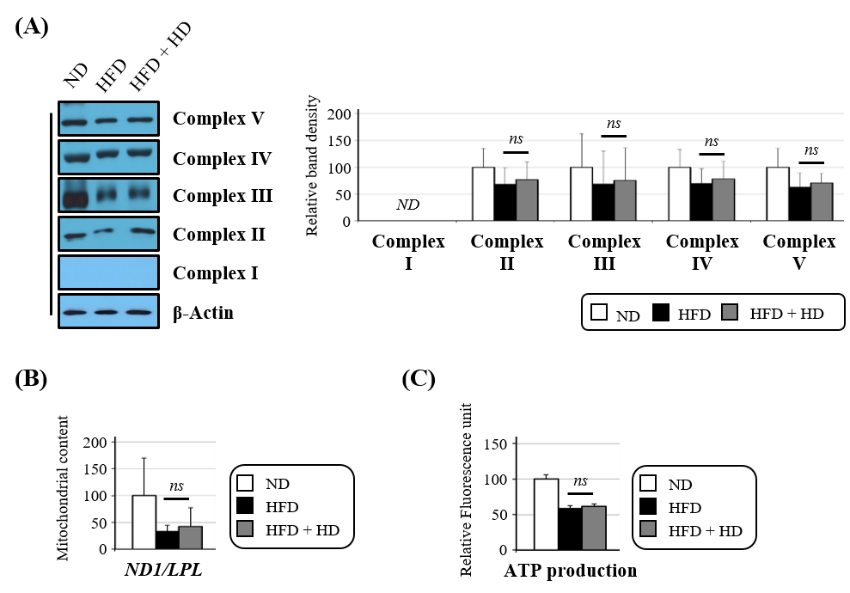


**Supplementary Figure 9.** HD6277 administration did not affect muscle mitochondrial performance in obese mice. (A) Mitochondrial OXPHOS proteins were determined by Western blotting. (B) Mitochondrial contents were measured by qPCR. (C) ATP production was calculated using a commercial ATP assay kit. *ND*, normal diet-fed mice; *HFD*, high-fat diet-fed mice; *HFD+HD*, HFD mixed with HD6277-fed mice; *ND*, no detect; *ns*, no significant. Error bars indicate the mean ± SD (ANOVA with post hoc *t*-test).

**Supplementary Tables**

**Supplementary table 1.** Antibody Lists used in the present study.

| **1^st^ Antibody** | **Source** | **Catalog No** | **Experiment** |
| --- | --- | --- | --- |
| phospho-Akt | Cell signaling | 9271S | Western Blotting |
| Total-Akt | Cell signaling | 9272S | Western Blotting |
| phospho-FOXO1A | Abcam | ab38501 | Western Blotting |
| Total-FOXO1A | Abcam | ab39670 | Western Blotting |
| Puromycin | Abcam | ab315887 | Western Blotting |
| MyoG | Santa Cruz | SC-12732 | Western Blotting |
| MyHC | Santa Cruz | SC-32732 | Western Blotting, ICC |
| Bax | Santa Cruz | SC-7480 | Western Blotting |
| Bcl-2 | Santa Cruz | SC-7382 | Western Blotting |
| cleaved Caspase-3 | Cell signaling | 9664S | Western Blotting |
| Ubiquitin | Cell signaling | 58395S | Western Blotting |
| Atrogin-1 | Abnova | PAB15627 | Western Blotting |
| MuRF1 | Abnova | PAB7497 | Western Blotting |
| beta-Actin | Santa Cruz | SC-47778 | Western Blotting |
| **2^nd^ Antibody** | **Source** | **Catalog No** | **Experiment** |
| Rabbit | Santa Cruz | PI-1000 | Western Blotting |
| Mouse | Santa Cruz | PI-2000 | Western Blotting |
| Goat | Santa Cruz | PI-9500 | Western Blotting |
| FITC-conjugated | Santa Cruz | SC-516140 | ICC |

**Supplementary table 2.** Primer sets used in the present study.

| **Target genes** | **Primer**  **Sequences** | **Product**  **size** | **Annealing temperature** |
| --- | --- | --- | --- |
| Nd1^3^ | F 5’-CCCATTCGCGTTATTCTT-3’ | 202bp | 56 ℃ |
|  | R 5’-AAGTTGATCGTAACGGAAGC-3’ |  |  |
| Lpl^3^ | F 5’-AGGTGGACATCGGAGAACTG-3’ | 157bp | 56 ℃ |
|  | R’ 5’-TCCCTAGCACAGAAGATGACC-3’ |  |  |
| PAX3^4^ | F 5’-GGGAACTGGAGGCATGTTTA-3’ | 151bp | 58 ℃ |
|  | R 5’-GTTTTCCGTCCCAGCAATTA-3’ |  |  |
| PAX7^5^ | F 5’-GTGCCCTCAGTGAGTTCGATTAGC-3’ | 169bp | 58 ℃ |
|  | R 5’-CCACATCTGAGCCCTCATCCA-3’ |  |  |
| Mef2c^6^ | F 5’-CGGTGTCGTCAGTTGTATGG-3’ | 168bp | 58 ℃ |
|  | R 5’-TGCAGTAGATATGCGGCTTG-3’ |  |  |
| Myf5^7^ | F 5’-AGGAAAAGAAGCCCTGAAGC-3’ | 151bp | 58 ℃ |
|  | R 5’-GCAAAAAGAACAGGCAGAGG-3’ |  |  |
| MyoD^8^ | F 5’-AGCACTACAGTGGCGACTCA-3’ | 201bp | 58 ℃ |
|  | R 5’-GCTCCACTATGCTGGACAGG-3’ |  |  |
| MyoG^9^ | F 5’-CTACAGGCCTTGCTCAGCTC-3’ | 200bp | 58 ℃ |
|  | R 5’-AGATTGTGGGCGTCTGTAGG-3’ |  |  |
| beta-Actin^10^ | F 5’-GCTCCTAGCACCATGAAGAT-3’ | 197bp | 56, 58 ℃ |
|  | R 5’-GTGTAAAACGCAGCTCAGTA-3’ |  |  |

**Reference**

1. Carter CS, Justice JN, Thompson L. Lipotoxicity, aging, and muscle contractility: does fiber type matter? *Geroscience* 2019;**41**:297-308.

2. Henrich CJ. A Microplate-Based Nonradioactive Protein Synthesis Assay: Application to TRAIL Sensitization by Protein Synthesis Inhibitors. *PLoS One* 2016;**11**:e0165192.

3. Medeiros DM. Assessing mitochondria biogenesis. *Methods* 2008;**46**:288-294.

4. Collins CA, Gnocchi VF, White RB, Boldrin L, Perez-Ruiz A, Relaix F, et al. Integrated functions of Pax3 and Pax7 in the regulation of proliferation, cell size and myogenic differentiation. *PLoS One* 2009;**4**:e4475.

5. Takegaki J, Sase K, Kono Y, Nakano D, Fujita T, Konishi S, et al. Intramuscular injection of mesenchymal stem cells activates anabolic and catabolic systems in mouse skeletal muscle. *Sci Rep* 2021;**11**:21224.

6. Ito N, Kii I, Shimizu N, Tanaka H, Takeda S. Direct reprogramming of fibroblasts into skeletal muscle progenitor cells by transcription factors enriched in undifferentiated subpopulation of satellite cells. *Sci Rep* 2017;**7**:8097.

7. Hewitt J, Lu X, Gilbert L, Nanes MS. The muscle transcription factor MyoD promotes osteoblast differentiation by stimulation of the Osterix promoter. *Endocrinology* 2008;**149**:3698-3707.

8. Scionti I, Hayashi S, Mouradian S, Girard E, Esteves de Lima J, Morel V, et al. LSD1 Controls Timely MyoD Expression via MyoD Core Enhancer Transcription. *Cell Rep* 2017;**18**:1996-2006.

9. Becker M, Joseph SS, Garcia-Carrizo F, Tom RZ, Opaleva D, Serr I, et al. Regulatory T cells require IL6 receptor alpha signaling to control skeletal muscle function and regeneration. *Cell Metab* 2023;**35**:1736-1751.e1737.

10. Zhou Z, Shen Y, Yin J, Xi F, Xu R, Lin D, et al. Matrix remodeling associated 7 promotes differentiation of bone marrow mesenchymal stem cells toward osteoblasts. *J Cell Physiol* 2019;**234**:18053-18064.
